# Pleiotropic phenotypic effects of the *TaCYP78A* family on multiple yield‐related traits in wheat

**DOI:** 10.1111/pbi.14385

**Published:** 2024-05-23

**Authors:** Meng Ma, Linnan Wu, Mengyao Li, Long Li, Lijian Guo, Deyan Ka, Tianxing Zhang, Mengdie Zhou, Baowei Wu, Haixia Peng, Zhaoxin Hu, Xiangli Liu, Ruilian Jing, Huixian Zhao

**Affiliations:** ^1^ College of Life Sciences Northwest A & F University Yangling Shaanxi China; ^2^ State Key Laboratory for Crop Stress Resistance and High‐Efficiency Production Northwest A & F University Yangling Shaanxi China; ^3^ State Key Laboratory of Crop Gene Resources and Breeding / Institute of Crop Sciences Chinese Academy of Agricultural Sciences Beijing China; ^4^ State Key Laboratory of Aridland Crop Science Gansu Agricultural University Lanzhou China; ^5^ College of Agronomy Northwest A & F University Yangling Shaanxi China; ^6^ College of Landscape Architecture and Art Northwest A & F University Yangling Shaanxi China; ^7^ Department of Electrical and Computer Engineering University of California San Diego La Jolla California USA

**Keywords:** wheat, yield‐related traits, synergistic improvement, trade‐offs, pleiotropy

## Abstract

Increasing crop yield depends on selecting and utilizing pleiotropic genes/alleles to improve multiple yield‐related traits (YRTs) during crop breeding. However, synergistic improvement of YRTs is challenging due to the trade‐offs between YRTs in breeding practices. Here, the favourable haplotypes of the *TaCYP78A* family are identified by analysing allelic variations in 1571 wheat accessions worldwide, demonstrating the selection and utilization of pleiotropic genes to improve yield and related traits during wheat breeding. The *TaCYP78A* family members, including *TaCYP78A3*, *TaCYP78A5*, *TaCYP78A16*, and *TaCYP78A17*, are organ size regulators expressed in multiple organs, and their allelic variations associated with various YRTs. However, due to the trade‐offs between YRTs, knockdown or overexpression of *TaCYP78A* family members does not directly increase yield. Favourable haplotypes of the *TaCYP78A* family, namely *A3/5/16/17Ap‐Hap II*, optimize the expression levels of *TaCYP78A3/5/16/17‐A* across different wheat organs to overcome trade‐offs and improve multiple YRTs. Different favourable haplotypes have both complementary and specific functions in improving YRTs, and their aggregation in cultivars under strong artificial selection greatly increase yield, even under various planting environments and densities. These findings provide new support and valuable genetic resources for molecular breeding of wheat and other crops in the era of Breeding 4.0.

## Introduction

Synergistic improvement of yield‐related traits (YRTs) is key to improving crop breeding effectiveness, but it is always hindered by the trade‐offs between YRTs (He *et al*., 2022; Wang *et al*., 2021). Some trade‐offs are caused by genetic linkage drag that can be solved by increasing recombination through conventional breeding (Huang *et al*., 2022; Voss‐Fels *et al*., 2017). However, some trade‐offs are caused by pleiotropy that cannot be easily overcome. For example, the ‘Green Revolution’ genes (*Rht*) reduce plant height (PH), but also decrease biomass, seedling vigour, and nitrogen‐use efficiency in many crops (Li *et al*., 2018; Liu *et al*., 2022); *TaCol‐B5* increases the number of tillers, spikes and spikelets and thus grain yield per plant (GYP), but also increases PH and the risk of lodging in wheat (Zhang *et al*., 2022). Therefore, the trade‐offs of pleiotropy remain to be resolved to break the yield ceiling.

Wheat (*Triticum aestivum* L.) is an important hexaploid (AABBDD, 2*n* = 42) cereal crop worldwide. Its YRTs, including spike number per plant (SN), grain number per spike (GNS), grain number per plant (GNP), and TGW, are complex traits controlled by quantitative trait loci or multiple genes. Trade‐offs between YRTs such as TGW and GNP or SN and GNS have been reported, and improving yield by increasing TGW has always been hindered by the trade‐off between TGW and GNP in wheat (Brinton and Uauy, 2019; Wiersma *et al*., 2001). The trade‐off effect greatly limits the synergistic improvement of YRTs and is difficult to overcome solely through conventional breeding (Molero *et al*., 2019).

Recently, the strategy of altering the expression pattern of certain genes in specific organs to overcome the trade‐offs has been adopted. For example, ectopic expression of *TaExpA6* (*ExpansinA 6*) in developing grain or localized overexpression of *TaCYP78A5* in developing ovary can increase TGW without reducing the GNP in wheat (Calderini *et al*., 2021; Guo *et al*., 2021). Practically, sustainable increases in wheat yield mainly rely on the synergistic improvement of YRTs (Li *et al*., 2022), which requires more understanding of the trade‐off effects and rational utilization of pleiotropy in wheat breeding.

**Figure 1:**
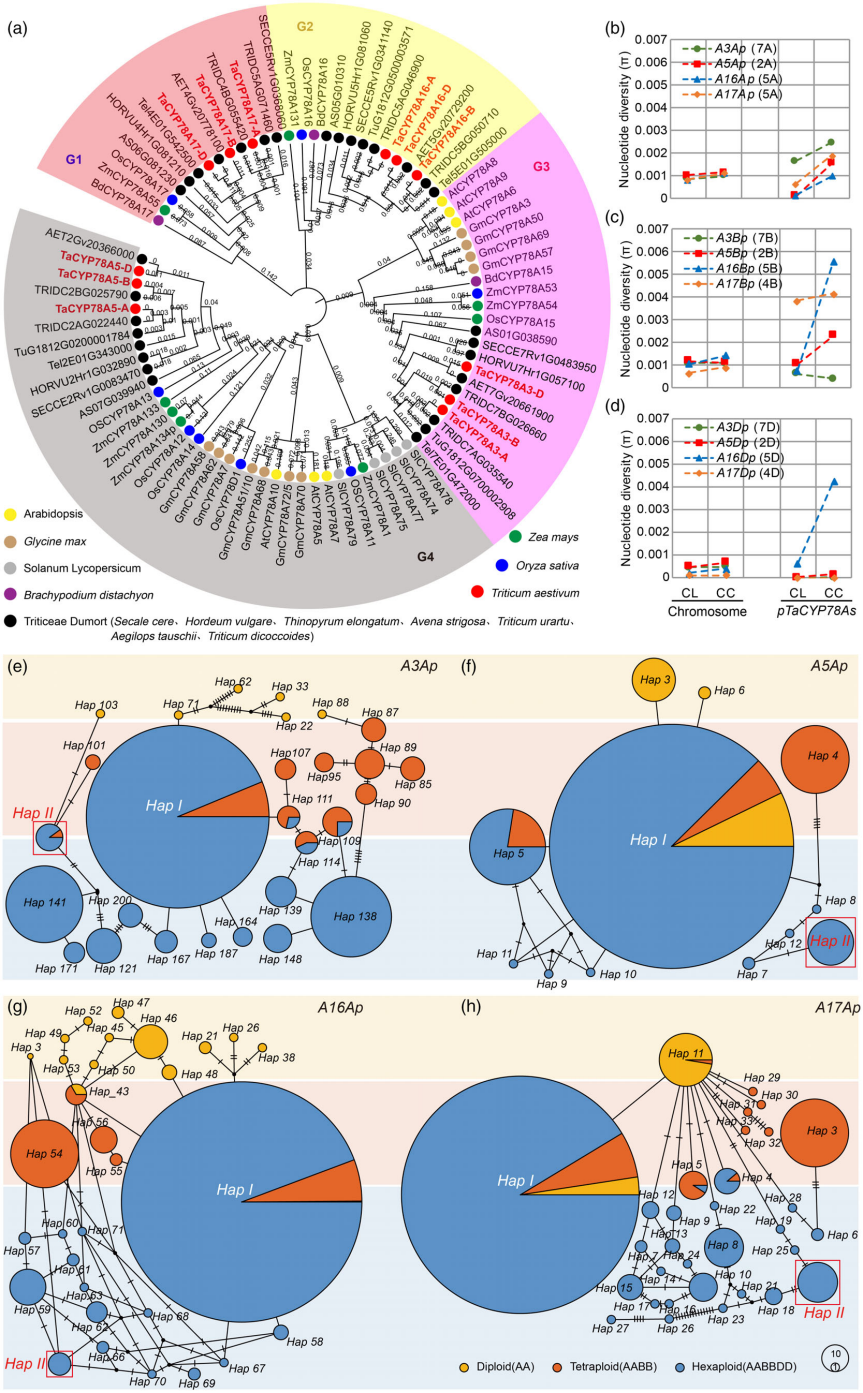
Phylogenetic, genetic diversity and haplotype analysis of *CYP78A* family in wheat. (a) Phylogenetic tree of *CYP78A* family in *Triticeae* and major plants. (b–d) Changes in the *π* value of the promoter regions of *TaCYP78A3/5/16/17‐A/B/D* during wheat breeding in China. Chromosome: average *π* value of each chromosome where the *TaCYP78A3/5/16/17‐A/B/D* located in. CL, Chinese Landraces; CC, Chinese Cultivars. *A3A/B/Dp*, *A5A/B/Dp*, *A16A/B/Dp*, and *A17A/B/Dp* represent the promoters of *TaCYP78A3/5/16/17‐A/B/D*, respectively. The number and letters within brackets indicated the chromosome number of *TaCYP78A3/5/16/17‐A/B/D*, respectively. (e–h) Evolution of the major haplotypes of *CYP78A* family during wheat polyploidization and domestication. A total of 1571 wheat accessions analysed contain 655 diploid, 125 tetraploid, and 791 hexaploid wheat accessions, including 465 cultivars. *Hap I* and *Hap II* indicate the two major haplotypes of *A3Ap*, *A5Ap*, *A16Ap*, and *A17Ap*. Each circle represents a haplotype, and the size of the circle represents the number of accessions with that haplotype. The black dashes on the line connecting two haplotypes represent the relative distance between them, with more dashes representing a greater relative distance.

The plant‐specific subfamily of cytochrome P450, *CYP78A*, has been regarded as a potential resource of genes for crop improvement due to its role in regulating the size of various plant organs (Anastasiou *et al*., 2007; Chakrabarti *et al*., 2013; Sun *et al*., 2017b; Ye *et al*., 2023; Zhou *et al*., 2022). Unfortunately, overexpression of *CYP78A* members (*CYP78As*) often does not result in increased GYP due to decreased seed setting (Adamski *et al*., 2009; Ma *et al*., 2015; Sotelo‐Silveira *et al*., 2013). In wheat, there are four members of the *TaCYP78A* family, namely *TaCYP78A3*, *TaCYP78A5*, *TaCYP78A16* and *TaCYP78A17* (*TaCYP78A3/5/16/17*), each with three homoeologs from the A, B, and D subgenomes, i.e., *TaCYP78A3/5/16/17‐A/‐B/‐D* (Figure 1a). Our previous studies have shown that *TaCYP78A3/5* regulates the size of grains and leaves (Guo *et al*., 2021; Ma *et al*., 2015; Zhou *et al*., 2022). However, how to utilize these pleiotropic effects to balance and improve multiple YRTs to achieve the goal of increasing grain yield remains a challenge.

Here, we identified four favourable haplotypes in the promoter region of *TaCYP78A3/5/16/17‐A* (*A3/5/16/17‐Ap*) based on the allelic variations of 1571 wheat accessions worldwide. Favourable haplotypes of *A3/5/16/17‐Ap* balance and improve multiple YRTs, and the improvement of YRTs in wheat breeding practices promotes the stacking of multiple favourable haplotypes into cultivars, thereby significantly improving yield. These findings provide support for high‐yield molecular breeding of wheat and even other crops.

## Results

### Phylogeny and genetic diversity of the *TaCYP78A* family in wheat

Phylogenetic analysis of CYP78A proteins from *Triticeae* (Vonbothmer *et al*., 1992) and other major crops, as well as *Arabidopsis*, showed that all CYP78As from different species could be clustered into four groups (G1–G4) (Figure 1a). CYP78As in the G1 and G2 groups were unique to *Poaceae* species, while CYP78As in the G3 and G4 groups were common to all the species analysed. In wheat, there are four members, i.e., TaCYP78A3/5/16/17, each having three homoeologs from the A, B, and D subgenomes (Figure 1a). The conserved heme domain FXXGXRXCXG is the main indicator for the identification of P450 (Chapple, 1998), and its sequence characteristics determine the group to which this CYP78A belongs (Figure S1 and Table S1).

To estimate the genetic diversity of *CYP78As* in wheat, we surveyed the allelic variations in both the coding and promoter regions of *TaCYP78A3/5/16/17* based on genomic data of 1571 accessions of the genus *Triticum* L. worldwide (Avni *et al*., 2017; Guo *et al*., 2020; He *et al*., 2019; Ma *et al*., 2021; Singh *et al*., 2019) (Tables S2 and S3). The results showed that significant changes occurred in the nucleotide diversity of the *TaCYP78A3/5/16/17‐A* promoters during wheat polyploidization and domestication processes; especially in wheat breeding in China, it was most abundant (Figure 1b–d and Figures S2–S4), which is consistent with artificial hybridization promoted efficient recombination in wheat breeding, thereby increasing the genetic diversity of the selection‐targeted regions (Hao *et al*., 2020). The ratios of nucleotide diversity of Chinese cultivars (CC) to Chinese landraces (CL) (*π*_CC_/*π*_CL_) at *A3/5/16/17Ap* (ranging from 1.5 to 13.2) were higher than the *π*_CC_/*π*_CL_ at the chromosome where *TaCYP78A3/5/16/17* located in (ranging from 1.1 to 1.3) (Figure 1b). *Tajima's D* test showed significant differences in some *A3/5/16/17Ap* (ranging from −0.94 to 2.79) between CC and CL (Table S2). Collectively, the diversity of *A3/5/16/17Ap* increased under selection during the wheat breeding process.

### Allelic variations of *A3/5/16/17Ap* in wheat are associated with multiple YRTs

**Figure 2:**
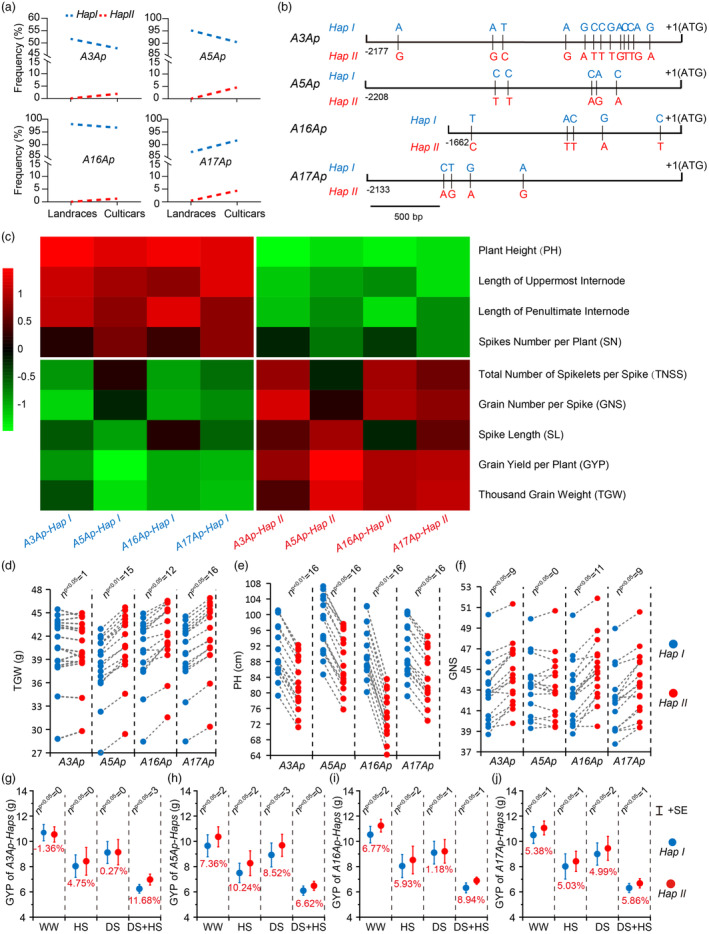
Correlation analysis between allelic variations of *TaCYP78A3/5/16/17‐Ap* and yield‐related traits in wheat. (a) Characterization of the haplotypes of *TaCYP78A3/5/16/17‐A* based on allelic variations in their promoter regions. *A3Ap*, *A5Ap*, *A16Ap*, and *A17Ap* represent the promoters of *TaCYP78A3/5/16/17‐A*, respectively. *Hap I* and *Hap II* indicate the two haplotypes in *A3Ap*, *A5Ap*, *A16Ap*, and *A17Ap*. (b) The frequency of the haplotypes of *TaCYP78A3/5/16/17‐A* in landraces (*n* = 209) and cultivars (*n* = 483). (c) Correlation between the *TaCYP78A3/5/16/17‐A* haplotypes and the yield‐related traits (YRTs) in wheat. (d–f) Statistical analysis of thousand grain weight (TGW, d), plant height (PH, e), and grain number per spike (GNS, f) of wheat accessions with different haplotypes in *A3/5/16/17Ap*. A point represents the average of phenotypic data from wheat accessions with different haplotypes of *TaCYP78A3/5/16/17‐Ap* at one environmental site. (g–j) Statistical analysis of the grain yield per plant (GYP) of wheat accessions with different haplotypes in *A3/5/16/17Ap* at different environmental sites. DS, drought stress at 3 environmental sites; DS + HS, drought and heat stress at 3 environmental sites; HS, heat stress at 5 environmental sites; WW, well water at 5 environmental sites. Phenotypic data were obtained from 323 wheat accessions planted at 16 environmental sites, and at least 10 plants of individual accessions were measured for each trait in each environment. “*n*” represents the numbers of environmental site where there was a significant difference in each YRT between accessions with *Hap I* and *Hap II* of *A3Ap*, *A5Ap*, *A16Ap*, and *A17Ap*. The number of wheat accessions with *Hap I* and *Hap II* of *A3Ap*, *A5Ap*, *A16Ap*, and *A17Ap* were 246/77, 58/265, 266/57, and 232/91, respectively. *P* < 0.05 and *P* < 0.01 by Student's *t*‐test.

Based on the allelic variations of the 1571 accessions mentioned above, we performed haplotype analysis of *A3/5/16/17Ap* and found that wheat accessions (*n* = 791) had the most abundant types of *A3/5/16/17Ap* haplotypes (Table S3). Phylogenetic analysis showed that the haplotypes of *A3/5/16/17Ap* in wheat could be divided into two groups; within each group, the number of cultivars with haplotypes *A3/5/16/17Ap‐Hap I* or *‐Hap II* was the highest (Figures S5 and S6). Moreover, the *A3/5/16/17Ap‐Hap I* initially emerged in diploid or tetraploid wheat and were widely distributed as ancient haplotypes in *Triticum* L. The *A3/5/16/17Ap‐Hap II* primarily appear in modern wheat cultivars and 10+ wheat genomic lines (Walkowiak *et al*., 2020) (Figure 1e–h; Figures S6 and S7), suggesting that *A3/5/16/17Ap‐Hap II* were new haplotypes developed in wheat breeding. Correspondingly, the frequency of cultivars with *A3/5/16/17Ap‐Hap I* decreased, but the frequency of cultivars with *A3/5/16/17Ap‐Hap II* increased compared to landraces during the wheat breeding process (Figure 2a). These indicated that *A3/5/16/17Ap‐Hap I* had been widely used in past wheat breeding practices, while *A3/5/16/17Ap‐Hap II* had been appreciated by many breeders worldwide during modern wheat breeding.

There are 13, 5, 5, and 4 SNPs between *A3/5/16/17Ap‐Hap I* and *‐Hap II*, respectively (Figure 2b), resulting in differences in several cis‐regulatory elements between them (Table S4). *TaCYP78A3/5/16/17‐A* were widely expressed in various wheat organs (Figure S8), and many QTLs associated with YRTs, including GW, PH, and GYP, overlapped with the loci of *A3/5/16/17Ap* (Figure S9). These suggested that the allelic variations of *A3/5/16/17Ap* may be associated with multiple YRTs. To test this speculation, we performed association analysis between the haplotypes of *A3/5/16/17Ap* from 323 wheat accessions (including 273 modern cultivars, 36 advanced lines and 14 landraces, Table S5) and their YRTs at 16 environmental sites. The results showed that the allelic variations in *A3/5/16/17Ap* were significantly (*P* < 0.05) associated with TGW, PH, GNS, GYP, spike length (SL), total number of spikelets per spike (TNSS) and SN (Figure 2c, Figure S10). Specifically, *A5/16/17Ap‐Hap II* were significantly associated with the increased TGW and GYP, *A3/16/17Ap‐Hap II* were significantly associated with the increased GNS and GYP, and all the *A3/5/16/17Ap‐Hap II* were associated with the decreased PH and SN. On the contrary, *A5/16/17Ap‐Hap I* were significantly associated with the decreased TGW and GYP, *A3/16/17Ap‐Hap I* were significantly associated with the decreased GNS and GYP, and all the *A3/5/16/17Ap‐Hap I* were associated with the increased PH and SN (Figure 2c–f, Figure S11). Therefore, *A3/5/16/17Ap‐Hap II* are considered as favourable haplotypes and *A3/5/16/17Ap‐Hap I* are considered as unfavourable haplotypes for high yield. Correspondingly, even under abiotic stresses, the accessions with *A3/5/16/17Ap‐Hap II* had higher GYP than the accessions with *A3/5/16/17Ap‐Hap I* at most environmental sites (increased by 0.27%–11.68%) (Figure 2g–j).

### Allelic variations in the promoters of *TaCYP78A3/5/16/17‐A* lead to their differential expression across wheat organs

**Figure 3:**
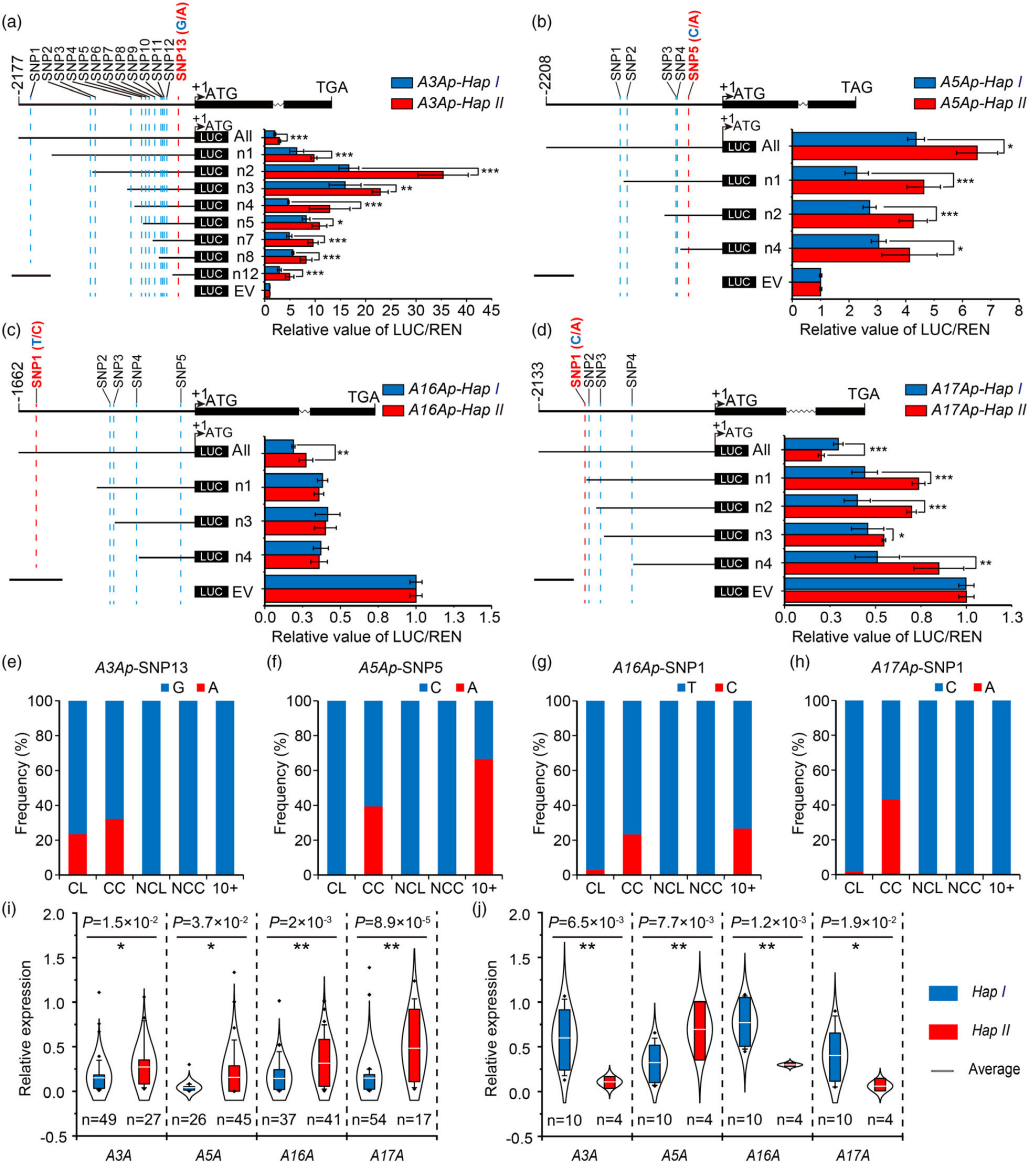
The effects of allelic variations in the promoters of *TaCYP78A3/5/16/17‐A* on their activity. (a–d) Analysis of the promoter activities of *TaCYP78A3/5/16/17Ap‐Hap l* and *‐Hap II* (named as *A3/5/16/17Ap‐Hap I* and *‐Hap II* for simplicity, respectively) by promoter truncation treatments (*n* = 3). The SNPs marked in red are the key allelic variation sites affecting the promoter activities of the haplotypes of *TaCYP78A3/5/16/17‐Ap*. EV, pGreen II 0800‐Luc empty vector; LUC, firefly luciferase; REN, renilla luciferase. (e–h) Distribution frequencies of the key allelic variation sites (SNPs marked in red or blue in a–d panels) of individual haplotypes of *TaCYP78A3/5/16/17‐Ap* in different wheat populations. (i, j) The expression levels of *TaCYP78A3/5/16/17‐A* in grains (i) and stems (j) of wheat accessions with different haplotypes of *TaCYP78A3/5/16/17‐Ap* (*n* accessions × 3 biological replicates). Errors are shown as ± SE (Standard Error); *P < 0.05, **P < 0.01 by Student's *t*‐test.

We evaluated the promoter activities of *A3/5/16/17Ap‐Hap I* and *‐Hap II* by dual‐luciferase assay in tobacco and found that the promoter activities of *A3/5/16/17Ap‐Hap II* were higher than those of *A3/5/16/17Ap‐Hap I in vitro* (Figure 3a–d). By promoter truncation experiments, we further identified the key SNPs in *A3/5/16/17Ap‐Hap I* and *‐Hap II*, namely *A3Ap‐SNP13* (−192 bp, G/A), *A5Ap‐SNP5* (−423 bp, C/A), *A16Ap‐SNP1* (−1493 bp, T/C), and *A17Ap‐SNP1* (−1283 bp, C/A), that resulted in differential promoter activities between *A3/5/16/17Ap‐Hap I* and *‐Hap II* (Figure 3a–d). These key SNPs of *A3/5/16/17Ap‐Hap I* were widely distributed in landraces and cultivars worldwide, whereas those of *A3/5/16/17Ap‐Hap II* were mainly distributed in a few CC and 10+ Wheat Genomes lines (Figure 3e–h). This is the same as the distribution pattern of *A3/5/16/17Ap‐Hap I* and *‐Hap II* in different wheat populations (Figure S6).

We further determined the expression levels of *TaCYP78A3/5/16/17‐A* in different organs of the wheat accessions with *A3/5/16/17Ap‐Hap I* or *‐Hap II*, and found that the accessions with *A3/16/17Ap‐Hap II* had higher expression levels of *TaCYP78A3/16/17‐A* in the grains but lower expression levels in the stems than those with *A3/16/17Ap‐Hap I*. Except for the accessions with *A5Ap‐Hap II*, which have higher expression levels in both grains and stems than the accessions with *A5Ap‐Hap I* (Figure 3i,j). Moreover, the accessions with *A3/5/16/17Ap‐Hap I* or *‐Hap II* had diversified expression levels of *TaCYP78A3/5/16/17‐A* in the spikes (Figure S12), which is consistent with the fact that *A3/5/16/17Ap‐Hap I* and *‐Hap II* were significantly associated with different spike traits (Figure S11). Together, allelic variations of *A3/5/16/17Ap* caused the differential expression levels of *TaCYP78A3/5/16/17‐A* across wheat organs, which appear to be closely related to YRTs.

### The expression levels of *TaCYP78A3/5/16/17‐A* affect multiple YRTs

**Figure 4:**
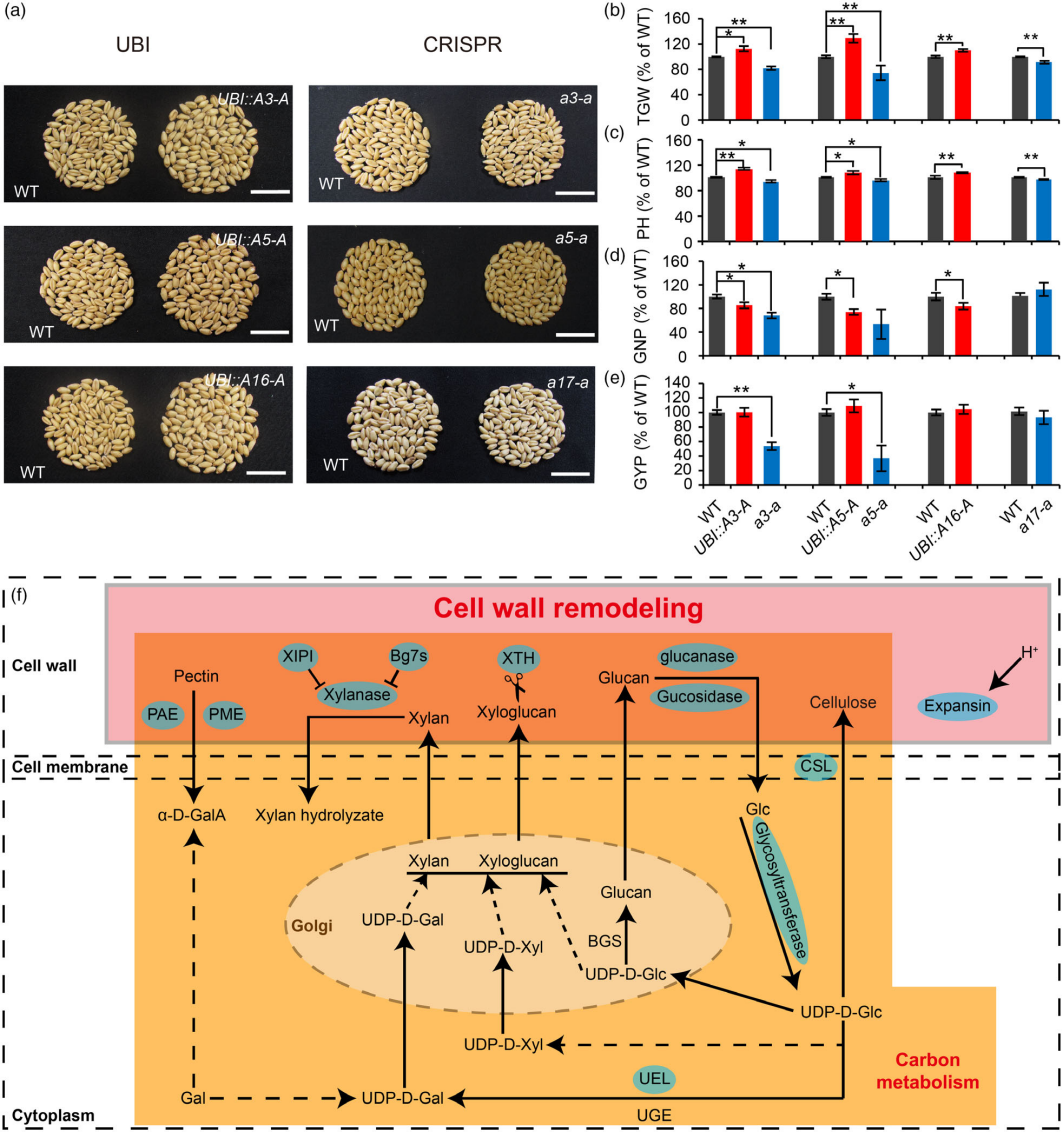
The expression levels of *TaCYP78A3/5/16/17‐A* affect the organ size and yield‐related traits of wheat. (a) Comparison of 100 grains from wild type (WT), *TaCYP78A3/5/16‐A* overexpressing lines (named as *UBI::A3/5/16‐A* for simplicity), and the loss‐of‐function mutants of *TaCYP78A3/5/17‐A* (named as *a3/5/17‐a* for simplicity). Bars = 2 cm. (b–e) Statistical analysis of thousand grain weight (TGW, b), plant height (PH, c), grain number per plant (GNP, d), and grain yield per plant (GYP, e) of different wheat genotypes (*n* > 10). Errors are shown as ± SE (standard error); *P < 0.05, **P < 0.01 by Student's *t*‐test. (f) Common differentially expressed genes (CDEGs) among the plants overexpressing *TaCYP78A3‐A*, *TaCYP78A5‐A*, or *TaCYP78A17‐A* are mainly involved in cell wall remodelling and polysaccharide metabolism. Blue ovals mark CDEGs, and squares represent pathways. α‐D‐Galacturonic acids; Bg7s, Basic 7S globulin; BGS, Beta‐glucans synthase; CSL, Cellulose synthase‐like protein; PAE, Pectin acetylesterase; PME, Pectin methylesterases; UDP‐D‐Xyl, UDP‐D‐xylose; UDP‐D‐Glc, UDP‐D‐glucose; UDP‐D‐Gal, UDP‐D‐galactose; α‐D‐GalA, UEL, UDP‐glucose 4‐epimerase‐like; UGE, UDP‐glucose 4‐epimerase; XIPI, Xylanase inhibitor protein 1; XTH, Xyloglucan endotransglucosylase/hydrolase.

To determine the effects of the expression levels of *TaCYP78A3/5/16/17‐A* on wheat YRTs, we generated transgenic wheat lines overexpressing *TaCYP78A3/5/16‐A* driven by the constitutive *Ubiquitin* promoter (*pUBI*) and loss‐of‐function mutants of *TaCYP78A3/5/17‐A* through the CRISPR‐Cas9 system (Figure S13). *TaCYP78A3/5/16‐A* overexpressing plants showed a significant increase in TGW (by 10.1%–29.2%), PH (by 6.9%–12.8%), and SL (by 8.5%–11.4%) compared with wild type (WT) plants (Figure 4a–e, Figure S14). In contrast, loss‐of‐function mutants of *TaCYP78A3/5/17‐A* had significantly reduced TGW (by 8.6%–25.5%) and PH (by 3.4%–16.7%) compared with WT plants (Figure 4a–e). In addition, changes in the expression levels of *TaCYP78A3/5/16/17‐A* led to changes in spike‐related traits such as SL, GNS, TNSS, and SN (Figure S14). Surprisingly, both overexpression and knockout of *TaCYP78A3/5/16/17‐A* led to a decrease in GNP (Figure 4d). Ultimately, overexpression or knockout of *TaCYP78A3/5/16‐A* did not increase GYP due to the trade‐off between TGW and GNP (Figure 4e and Figure S14).

To reveal the mechanism by which *TaCYP78A3/5/16/17‐A* regulate wheat YRTs, we sequenced and analysed the transcriptomes of grain samples from *TaCYP78A3/5/17‐A* overexpressing plants and WT plants, and identified 238 common differentially expressed genes (CDEGs) between them (*q*‐value <0.05, fold change >1.5) (Figure S15 and Table S6). These CDEGs were mainly enriched in phytohormone signalling and carbon metabolism pathways (Figure S16 and Table S6). Further analysis revealed that 32% (23/73) of the CDEGs related to carbon metabolism‐related processes were involved in cell wall polysaccharide metabolism, which is directly related to cell wall remodelling processes and is regulated by phytohormone signalling (Figure 4f, Figure S16 and Table S6) (Schoenaers *et al*., 2018; Yang *et al*., 2023). We further investigated the organ sizes and cytological characteristics of wheat accessions with different number of *A3/5/16/17Ap‐Hap II*, and found that their organ size was positively correlated with the number of epidermal cells (Figures S17 and S18). In general, the expression levels of *TaCYP78A3/5/16/17‐A* affect multiple YRTs, which may be involved in cell wall remodelling regulated by phytohormone signals in wheat.

### Favourable haplotypes have aggregation effects on the improvement of yield and related traits

**Figure 5:**
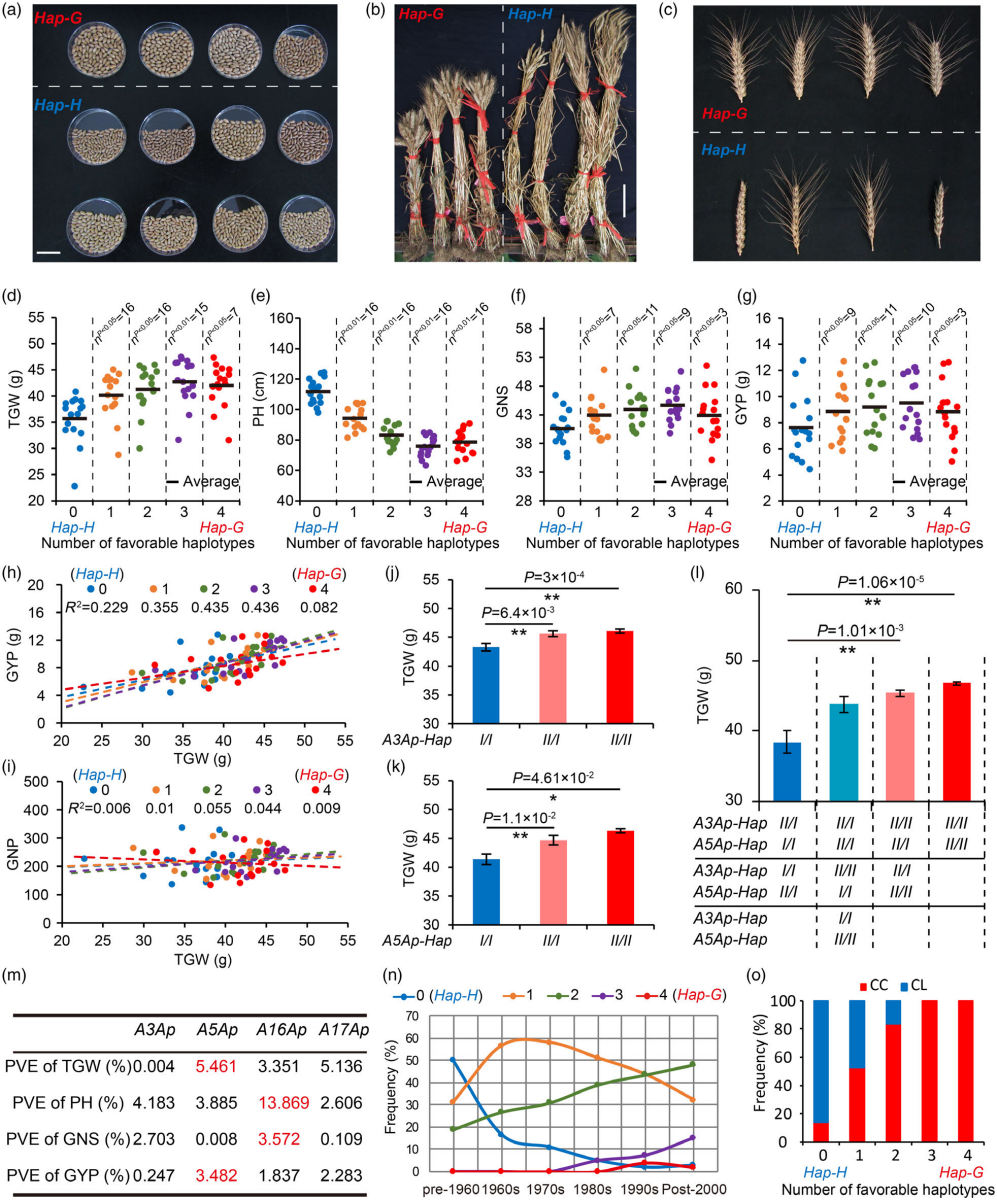
Aggregation effects of the favourable haplotype of *TaCYP78A3/5/16/17‐Ap* on yield‐related traits of wheat. (a–c) Comparison of 100 grains (a), plant height (b), and spike type (c) of wheat accessions with *Hap‐H* and *Hap‐G*. *Hap‐H/−G*: Wheat accessions with zero/four favourable haplotype of *TaCYP78A3/5/16/17‐Ap* (*A3/5/16/17Ap‐Hap II*). Bars in (a), (b), and (c) indicate 3, 20, and 5 cm, respectively. (e–g) The relationship between the numbers of *A3/5/16/17Ap‐Hap II* and TGW (d), PH (e), GNS (f), and GYP (g) at 16 environmental sites. “n” represents the numbers of environmental sites with significant differences in this YRT between *Hap‐H* and other genotypes. (h, i) Correlation between TGW and GYP (h) or between TGW and GNP (i) of wheat accessions with different numbers of *A3/5/16/17Ap‐Hap II*. (j, k) TGW of Near‐Isogenic Lines (NILs) with homozygous or heterozygous *A3Ap‐Hap I*/*II* or *A5Ap‐Hap I*/*II* (*n* > 4). *A3Ap‐Hap I*/*II* and *A5Ap‐Hap I*/*II* indicate the different haplotypes of *TaCYP78A3‐Ap* and *TaCYP78A5‐Ap*, respectively. (l) Aggregation effects of *A3/5Ap‐Hap II* on TGW. The horizontal axis displays NILs with 1–4 *A3Ap‐Hap II* or *A5Ap‐Hap II* from left to right. (m) The phenotypic variance explanation (PVE) of allelic variations of *A3/5/16/17Ap* on different yield‐related traits. (n) Frequency changes of different number of *A3/5/16/17Ap‐Hap II* over decades in modern cultivars in China. Overall 14, 33, 47, 34, 60, and 123 accessions were released in the pre‐1960s, 1960s, 1970s, 1980s, 1990s, and post‐2000, respectively. (o) The proportion of Chinese Landraces (CL, *n* = 126) to Chinese Cultivars (CC, *n* = 352) in accessions with different numbers of *A3/5/16/17Ap‐Hap II*. Phenotypic data in d–i and m panels were from 323 wheat accessions at 16 environmental sites. Individual points in d–i panels represent the average of phenotypic data from 25, 140, 128, 26, and 4 wheat accessions with 0–4 *A3/5/16/17Ap‐Hap II* at one environmental site, respectively. *P < 0.05, **P < 0.01 by Student's *t*‐test.

Considering that the favourable haplotypes *A3/5/16/17Ap‐Hap II* had similar but not identical effects on the improvement of wheat YRTs (Figure 2), we further analysed their aggregation effects in the 323 wheat accessions mentioned above. We first divided these accessions into five groups, with the same number of *A3/5/16/17Ap‐Hap II* for each accession in each group (Table S7). We then compared the YRTs among these groups and found that as the number of *A3/5/16/17Ap‐Hap II* increased, the TGW, GNS, and GYP of accessions in this group showed an increasing trend at 16 environmental sites, while their PH and SN showed a decreasing trend (Figure 5a–g and Figure S19). In particular, the increase in GNS offset the GNP loss caused by the decrease in SN, thereby maintaining the GNP unchanged (Figure S19). In this way, GNP was decoupled from TGW, unlike in the transgenic lines where GNP decreased with increasing TGW (Figure 4d). Association analysis showed that TGW was positively related to GYP but not to GNP in different groups (Figure 5h,i). These results indicated that the aggregation of *A3/5/16/17Ap‐Hap II* was beneficial for the synergistic improvement of wheat YRTs, ultimately improving GYP by overcoming the trade‐off between TGW and GNP.

We further investigated the aggregation effects of *A3/5/16/17Ap‐Hap II* on TGW using near‐isogenic lines (NILs) with different haplotypes of *A3/5‐Ap*, and found that the TGW was significantly higher in NILs with *A3/5Ap‐Hap II* than in NILs with *A3/5Ap‐Hap I* (Figure 5j,k). Detailed analysis of the TGW between homozygous and heterozygous plants of these NILs showed that the TGW of heterozygous plants (*I/II*) was smaller than that of homozygous plants with *A3/5Ap‐Hap II* (*II/II*) but larger than that of homozygous plants with *A3/5Ap‐Hap I* (*I/I*), suggesting that *A3/5Ap‐Hap II* were semi‐dominant alleles in regulating TGW (Figure 5j–l). Moreover, the aggregation of *A3Ap‐Hap II* and *A5Ap‐Hap II* in individual wheat accessions resulted in an upward trend of TGW (Figure 5l), which is consistent with the increase in TGW caused by the gradual aggregation of *A3/5/16/17Ap‐Hap II* in different groups of wheat accessions (Figure 5d).

**Figure 6:**
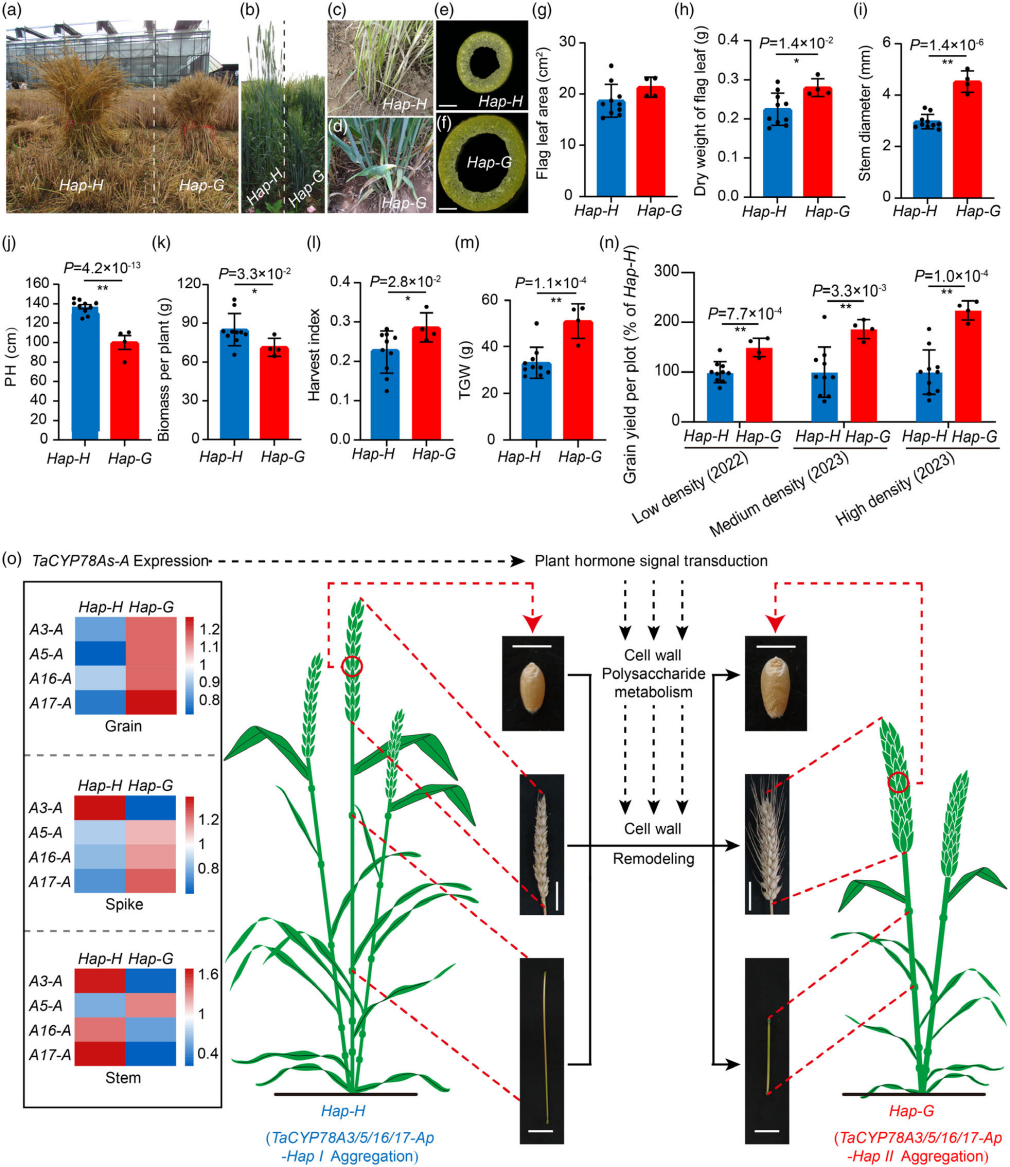
Aggregation of the favourable haplotypes of *TaCYP78A3/5/16/17‐Ap* improve the yield and related traits of wheat. (a) Yield test of accessions with *Hap‐H* or *Hap‐G* in plots. *Hap‐H*: Genotypes with zero favourable haplotype of *TaCYP78A3/5/16/17‐Ap* (*A3/5/16/17Ap‐Hap II*); *Hap‐G*: Genotypes with four *A3/5/16/17Ap‐Hap II*. Ten accessions with *Hap‐H* and four accessions with *Hap‐G* grown in the 4‐m^2^ plots were tested. (b–f) Comparison of plant height (b), tiller number (c and d), and stem cross‐sections (e and f) of accessions with *Hap‐H* or *Hap‐G* at the flowering stage. Bar = 1 mm. (g–m) Statistical analysis of flag leaf area (g), dry weight of flag leaf (h), stem diameter (i), PH (j), biomass per plant (k), harvest index (l) and TGW (m) of accessions with *Hap‐H* or *Hap‐G* in the yield test (*n* = 4 or 10 accessions × 15 biological replicates). (n) Statistical analysis of grain yield per plot of accessions with *Hap‐H* or *Hap‐G* in the yield test (*n* = 4 or 10 accessions × 3 plots; 200 (low density planting in 2022), 400 (middle density planting in 2023), and 800 (high density planting in 2023) plants were planted in a single plot of 4 m^2^ in the field). Individual points in g–n panels represent the average of phenotypic data from one wheat accession with *Hap‐H* or *Hap‐G*. (o) A model of the haplotypes of *TaCYP78A3/5/16/17‐Ap* regulating wheat YRTs. The expression data used in the heatmap were derived from the mean expression levels of *TaCYP78A3/5/16/17‐A* in individual organs of four accessions with *Hap‐G* or 10 accessions with *Hap‐H* (three biological replicates). Bars = 0.5, 3, and 5 cm in grain, spike, and stem morphology, respectively. Values indicate means ± SE. *P < 0.05, **P < 0.01 by Student's *t*‐test.

Next, we compared the yield and related traits of accessions with four *A3/5/16/17Ap‐Hap II* (named as *Hap‐G*, *n* = 4) with those of accessions with zero *A3/5/16/17Ap‐Hap II* (named as *Hap‐H*, *n* = 10) in detail, and found that the accessions with *Hap‐G* had comprehensive advantages over the accessions with *Hap‐H*. The accessions with *Hap‐G* had thicker stem, heavier leaf, limited SN, lower PH, more GNS, and higher TGW and harvest index than the accessions with *Hap‐H* (Figure 6a–m and Figure S20). Unexpectedly, the grain yield per plot of the accessions with *Hap‐G* increased by 49.59%, 86.39%, and 123.94% compared to the accessions with *Hap‐H* under low density (50 plants/m^2^), medium density (100 plants/m^2^), and high density (200 plants/m^2^) planting conditions in the field, respectively (Figure 6n). Combined with the fact that the aggregation of *A3/5/16/17Ap‐Hap II* in accessions could enhance their GYP even under drought and high temperature stress (Figure S19), we concluded that the aggregation of *A3/5/16/17Ap‐Hap II* endowed wheat with stable and high yield in various environments by improving multiple YRTs.

### The aggregation effects arise from the complementary and specific effects of individual favourable haplotypes

To understand the cause of aggregation effects, we investigated the contribution of different *A3/5/16/17Ap‐Hap II* to the improvement of YRTs. *A3Ap‐Hap II* and *A16Ap‐Hap II* had higher phenotypic variance explanation (PVE) for PH and GNS but lower PVE for TGW and GYP, while *A5Ap‐Hap II* and *A17Ap‐Hap II* had higher PVE for TGW and GYP but lower PVE for PH and GNS (Figure 5m), suggesting that *A3/5/16/17Ap‐Hap II* had complementary effects on the improvement of wheat YRTs. This was consistent with the complementary expression levels of *TaCYP78A3/5/16/17‐A* in different organs (Figures S8 and S21), and supported a previous speculation that *CYP78As* are functionally differentiated through organ‐specific expression (Nobusawa *et al*., 2021).

We further compared the expression levels of *TaCYP78A3/5/16/17‐A* in the accessions with *Hap‐G* or *Hap‐H*, and found that the accessions with *Hap‐G* showed higher expression levels of *TaCYP78A3/5/16/17‐A* in grains, spikes, and leaves, but lower expression levels in stems than those with *Hap‐H* (Figure 6o and Figure S22). The accessions with *Hap‐G* or *Hap‐H* showed a complementary expression levels of *TaCYP78A3/5/16/17‐A* in these organs (Figure S22). Taken together, these results confirmed that the aggregation effects stemmed from the complementary effects of individual favourable haplotypes of *A3/5/16/17‐Ap* on the improvement of wheat YRTs, which were closely related to the complementary expression levels of *TaCYP78A3/5/16/17‐A* in different organs.

### The improvement of YRTs associated with the aggregation of favourable haplotypes into cultivars

We investigated the aggregation of *A3/5/16/17Ap‐Hap II* in wheat breeding practices and found that accessions with few *A3/5/16/17Ap‐Hap II* had been gradually phased out, but accessions with multiple *A3/5/16/17Ap‐Hap II* have been increasing during the past 60 years of wheat breeding in China (Figure 5n and Figure S23), suggesting that accessions with multiple *A3/5/16/17Ap‐Hap II* had been favoured in modern wheat breeding in China. Moreover, during the past 60 years of wheat breeding in China, wheat improvement was mainly related to a gradual increase in TGW, GNS, GNP, and GYP and a gradual decrease in PH and SN (Figure S24). This had been proven to be an effective way to increase wheat production and was highly consistent with the aggregation effects of *A3/5/16/17Ap‐Hap II* on improving wheat yield potential (Figures 5a–g and 6o). With the improvement of these YRTs, the number of *A3/5/16/17Ap‐Hap‐II* in individual cultivars gradually increased (Figure 5n,o, Figure S24), which could explain 0.04%–56.35% of the phenotype variation of GYP at different planting environments (Figure S19). Notably, the frequency of specific genotypes with multiple *A3/5/16/17Ap‐Hap‐II* in cultivars fluctuated dramatically (Figure S23), suggesting that the selection of multiple *A3/5/16/17Ap‐Hap‐II* in the wheat breeding practices was mainly by targeting them as a group rather than targeting specific combinations or genotypes. These results indicated that during the past 60 years of wheat breeding practices in China, strong artificial selection on YRTs promoted the aggregation of *A3/5/16/17Ap‐Hap II* into individual cultivars, thereby improving wheat production.

## Discussion

Wheat is one of the world's most important staple crops, and its yield is directly related to human food security. With the increase in the world population and the frequent occurrence of extreme climates leading to the loss of wheat production, the issue of food security problem worldwide is becoming increasingly prominent. It is a practical and effective way to sustainably increase wheat yield by improving YRTs through combining the synergistic effects of multiple haplotypes of genome regions known to moderate yield attributes. However, due to the trade‐offs between YRTs, synergistic improvement of YRTs is a challenging task for wheat breeding (Brinton and Uauy, 2019; Wiersma *et al*., 2001; Xiao *et al*., 2022). Recently, a large number of studies have mainly focused on a few target YRTs, ignoring the trade‐offs between YRTs (Visscher and Yang, 2016). To date, little is known about how to overcome the trade‐offs between YRTs and achieve synergistic improvement of YRTs to increase yield. Here, we found that *TaCYP78A3/5/16/17‐A* positively regulated multiple organ sizes, and the favourable variations in their promoters could optimize their expression levels across organs, thereby balancing and improving multiple YRTs and achieving the improvement of YRTs (Figures 2, 3, 4). The favourable haplotypes of *A3/5/16/17Ap* had pleiotropic and aggregation effects on the improvement of YRTs, which greatly improved wheat yield and related traits even under various planting environments and densities (Figures 5, 6, Figure S19). Strong artificial selection on wheat YRTs in wheat breeding practices promoted the favourable haplotypes of *A3/5/16/17Ap* stacking in individual cultivars, leading to more modern cultivars with improved plant type and increased yield (Figures 5, 6). These findings provide new support and valuable genetic resources for “molecular design breeding and molecular aggregation breeding” of wheat and other crops in the era of Breeding 4.0.

*CYP78As* are widely distributed in plants and have similar functions in regulating organ size (Figure 1) (Anastasiou *et al*., 2007; Chakrabarti *et al*., 2013; Sun *et al*., 2017b; Zhou *et al*., 2022, 2023). *CYP78As* are typical pleiotropic genes and rely on a downstream mobile hormone‐like signalling molecule to produce a non‐cell‐autonomous growth‐promoting effect in the plant organs around the expression sites (Eriksson *et al*., 2010; Nobusawa *et al*., 2021; Zhou *et al*., 2022). Here, we found that *TaCYP78A3/5/16/17‐A* are widely expressed in various wheat organs and regulate organ growth by promoting their cell proliferation (Figure 4, Figures S8, S17 and S18). However, due to the trade‐off between TGW and GNP, both the loss‐of‐function and overexpression of *TaCYP78A3/5/16/17‐A* led to a reduction in GYP (Figure 4). Similar phenomena have also been observed in plants with overexpression or loss‐of‐function of *CYP78A5* or *CYP78A9* in *Arabidopsis* (Adamski *et al*., 2009; Sotelo‐Silveira *et al*., 2013; Zhao *et al*., 2018).

To overcome the trade‐off between TGW and GNP, we previously overexpressed *TaCYP78A5* locally in the developing ovaries, resulting in a significant increase in both TGW and GYP of *TaCYP78A5* overexpressing lines in wheat (Guo *et al*., 2021). However, the proportion of increase in GYP was much smaller than that of TGW, suggesting that there is still a certain trade‐off between TGW and GNP in *TaCYP78A5* overexpressing lines. In the present study, we found that the favourable haplotypes *A3/5/16/17Ap‐Hap II* showed similar attributes in improving multiple YRTs. In particular, wheat accessions with *A3/5/16/17Ap‐Hap II* showed optimized expression levels and complementary PVE of *TaCYP78A3/5/16/17‐A* across plant organs (Figures 3i,j, 6o; Figures S21, S22), thus leading to a balanced and synergistic improvement of multiple YRTs in wheat, including an increase in TGW and GNS without a decrease in GNP, as well as a decrease in PH and restriction of SN while still increasing grain yield (Figures 2, 5, 6). Similarly, the trade‐off between GNS and SN can be overcome by optimizing the expression levels of *IPA1* (*Ideal plant architecture 1*) (Song *et al*., 2022). Taken together, by optimizing the expression levels of pleiotropic genes across plant organs, it may be possible to overcome the trade‐off between YRTs caused by gene pleiotropy. Similar strategies have also been proven effective in crop stress resistance breeding (Gu *et al*., 2023; Mao *et al*., 2022; Zhang *et al*., 2020).

Crop domestication and breeding rely on the selection of pleiotropic yield‐related loci for synergistic improvement of YRTs (Doebley *et al*., 2006; Li *et al*., 2022; Sun *et al*., 2017a). Several pleiotropic yield‐related genes were cloned by QTL mapping, which greatly improved the application of marker‐assisted breeding and facilitated the genetic improvement of yield and related traits (Chen *et al*., 2022; Saini *et al*., 2022; Song *et al*., 2023). However, these loci and their candidate genes and roles remain mostly unknown in wheat. Here, we found that *TaCYP78A3/5/16/17‐A* are located in the interval of multiple QTLs related to yield traits in A subgenomes and have been strongly selected during wheat domestication and breeding (Figures S1–S4, S9), suggesting asymmetric subgenome selection in wheat breeding for high yield. This is in line with previous reports (Hao *et al*., 2020). Individual favourable haplotype of *A3/5/16/17Ap* showed both similarity and pleiotropy, specificity and complementarity, in improving YRTs, which led to their stacking exhibiting aggregation effects and significantly increasing yield even under various planting environments and densities (Figures 2, 5, 6; Figure S19). Probably due to their similarity and complementarity, the selection and utilization of multiple favourable haplotypes of *A3/5/16/17Ap* in the wheat breeding process were mainly directed towards them as a group rather than being restricted to a particular combination (Figure 5n,o and Figure S23), which also facilitated their stacking in more cultivars. As is well known that YRTs are the main targets of artificial selection during crop domestication and breeding (Doebley *et al*., 2006; Huang *et al*., 2022). Therefore, in modern wheat breeding practice, cultivars with multiple favourable haplotypes of *A3/5/16/17Ap* have been actively selected due to the synergistic improvement of YRTs (Figure 5). It has also been reported that a key SNP in the promoter of *CYP78A5* in tomato is associated with the domestication of fruit size. Furthermore, the rich genetic diversity of *CYP78A13* in japonica rice facilitates the improvement of TGW and yield compared to indica rice (Chakrabarti *et al*., 2013; Xu *et al*., 2015). Thus, stacking favourable variations of *CYP78As* can synergistically improve YRTs and has been favoured in crop breeding practices because it caters to the needs of plant breeders targeting the improvement of grain yield.

## Experimental procedures

### Phylogenetic analysis of *CYP78As*

We performed a multiple sequence alignment of *CYP78As* proteins from 13 species (including *A*. *thaliana*, *O*. *sativa*, *Glycine max*, *Zea mays*, *Brachypodium distachyon*, *Lycopersicon esculentum*, Secale cereal, *Hordeum vulgare*, *Thinopyrum elongatum*, *Avena strigosa*, *T*. *urartu*, *T*. *dicoccoides*, *Aegilops tauschii* and *T*. *aestivum*) using MUSCLE software (http://www.ebi.ac.uk/Tools/msa/muscle/). The phylogenetic tree was constructed with the MEGA 7 software using the neighbour‐joining statistical method with 1000‐replicate bootstraps (Kumar *et al*., 2016).

### Haplotype, SNP and sequence analysis

The coding and promoter sequences for *TaCYP78A3/5/16/17* from 1571 accessions of the genus *Triticum* were obtained from the WheatOmics 1.0 (http://202.194.139.32/) (Ma *et al*., 2021) and Wheat‐SnpHub‐Portal (http://wheat.cau.edu.cn/Wheat_SnpHub_Portal/) databaes (Wang *et al*., 2020). Sequence alignment and SNP detection were carried out by DNAMAN 8.0 software (http://www.lynnon.com/). Haplotype analysis was performed based on the DNA sequences of *TaCYP78A3/5/16/17* from 1571 accessions using PopART (http://popart.otago.ac.nz/index.shtml) (Leigh and Bryant, 2015). Key cis‐acting elements were predicted from the *TaCYP78A3/5/16/17* promoter sequences using PlantCARE software (http://bio‐informatics.psb.ugent.be/webtools/plantcare/html/). Genetic mapping of *TaCYP78A3/5/16/17‐A* was performed using MapChart software (Voorrips, 2002).

### Haplotype cloning and sequencing

Based on the results of the sequence analysis, the full‐length and truncated (5′‐end successive deletions) specific primer sequences of *TaCYP78A3/5/16/17‐Ap* haplotypes were designed using Primer Premier 5.0 software (http://www.premierbiosoft.com/). DNA samples from leaves of accessions with different *TaCYP78A3/5/16/17‐Ap* haplotypes were used as templates for cloning. Amplified products were sequenced and verified. The primers used are listed in Table S8.

### Growth conditions and phenotypic evaluation

To perform correlation analysis between haplotypes of *TaCYP78A3/5/16/17‐Ap* and YRTs, 323 wheat accessions were planted at Changping (116°13′ E; 40°13′ N) and Shunyi (116°56′ E; 40°23′ N) experimental stations of the Institute of Crop Science, CAAS, Beijing, over three years (from 2015 to 2017) to measure agronomic traits. Two water regimes, rain‐fed (drought stressed, DS) and well‐watered (WW) were established at each site. In addition, a greenhouse was constructed in Shunyi to simulate heat stress (HS). The 16 environments (E1 to E16) indicate the environments at Shunyi in 2015 under drought stress (DS), DS + heat stress (HS), well‐watered (WW), WW + HS; Shunyi in 2016 under DS, DS + HS, WW, WW + HS; Changping in 2016 under DS and WW; Shunyi in 2017 under DS, DS + HS, WW, WW + HS; Changping in 2017 under DS and WW, respectively. At least 10 plants of individual accessions were measured for each trait in each environment.

Accessions with *Hap‐G* or *Hap‐H* and near‐isogenic lines (NILs) were grown in the experimental field, Northwest A & F University, Yangling, Shaanxi, China (longitude 108°4′ E, latitude 34°17′ N) during the natural growing season from 2021 to 2022 and 2022 to 2023. For the yield test in the field, four accessions with *Hap‐G* and 10 accessions with *Hap‐H* were grown in 3 plots, each plot containing 200, 400 and 800 plants in a 4‐m^2^ field under low density (50 plants/m^2^), medium density (100 plants/m^2^), and high density (200 plants/m^2^) planting conditions, respectively. At least 15 plants of each accession were measured for agronomic traits. Stem diameter was the diameter of the second upper internode after heading. All plants were harvested at full maturity for yield measurements. To investigate the NILs with different *TaCYP78A3/5‐Ap* favourable haplotypes, we grew 20 rows, 20 plants per row, for a total of 400 plants in the experimental field and performed phenotypic analysis and genotype identification on 100 randomly selected 100 plants.

All transgenic lines were grown in a greenhouse with a 16/8 h light/dark cycle, a temperature range of 20–25/15–18 °C, and a humidity of 50%, and were watered as needed. The Supplementary Methods S1 provide a detailed introduction to the construction and identification of transgenic lines, as well as growth conditions and phenotype evaluation.

### RNA sequencing and data analysis

The RNA samples were isolated from the 2 mm ovaries of the *TaCYP78A3/5/17* transgenic lines as previously reported (Guo *et al*., 2021). The RNA samples that passed the quality control were sent to Gene Denovo (Guangzhou, China) for cDNA library construction and sequencing on the sequencing platform (Illumina HiSeq™ 2500), and the exact amount of each RNA sample was further assessed using Agilent 2100 Bio‐analyser (Agilent Technologies, Santa Clara, CA).

The differentially expressed genes (DEGs) between the transgenic lines and WT were identified (*q*‐value <0.05, fold change >1.5). All the DEGs were subjected to Jvenn (http://jvenn.toulouse.inra.fr/app/index.html) (Bardou *et al*., 2014) and the CDEGs were exported (Table S6). Gene Ontology (GO) and Kyoto Encyclopedia of Genes and Genomes (KEGG) enrichment of all detected CDEGs were performed as previously described (Chi *et al*., 2019). RNA extraction and expression analysis are described in detail in the Supplementary Methods S1.

### Cellular analyses

To observe cell characteristics, the epidermis of mature grains and stems from accessions with different *TaCYP78A3/5/16/17‐Ap* haplotypes were stained with toluidine blue as previously reported (Guo *et al*., 2021), and further observed using a Leica bio‐optical microscope (DM500). ImageJ software (National Institutes of Health) was used to measure the size and number of epidermal cells.

### Detection of promoter activity

Using the DNA from wheat accessions with different haplotypes of *TaCYP78A3/5/16/17‐Ap* as templates, the promoter sequence of *TaCYP78A3/5/16/17‐A* was amplified by PCR and inserted into the *pGreenII 0800‐LUC* vector using *KpnI* and *NcoI* restriction endonucleases. Primers are listed in Table S8. The plasmids were introduced into the *Agrobacterium tumefaciens* strain *GV3101* (*pSoup‐p19T*) and transformed into *Nicotiana benthamiana* leaves. After transformation, the tobacco plants were incubated at 23°C for 3 days. Fluorescent LUC and REN activities were detected using the Dual‐Luciferase Reporter Assay System (Promega). LUC activity was normalized to REN activity.

## Conflict of interest

The authors have not declared a conflict of interest.

## Supporting information


**Figure S1** Analysis of conserved domains of *CYP78A* family in plants.
**Figure S2** Phylogenetic and genetic diversity analysis of *CYP78A* family in plants.
**Figure S3** Changes of nucleotide polymorphisms of *TaCYP78A3/5/16/17* during wheat polyploidy and domestication.
**Figure S4** Changes of the *π* value of *TaCYP78A3/5/16/17* during the domestication in Non‐Chinese common wheat.
**Figure S5** Evolutionary analysis of different haplotypes of *TaCYP78A3/5/16/17‐Ap* in wheat.
**Figure S6** Evolutionary history and distribution of the haplotypes of *TaCYP78A3/5/16/17‐Ap*.
**Figure S7** Distribution of different haplotypes of *TaCYP78A3/5/16/17‐Ap* during wheat domestication.
**Figure S8** Expression profiles of *TaCYP78A3/5/16/17* in major organs of wheat.
**Figure S9** Known QTLs related to yield‐related traits in the upstream and downstream of *TaCYP78A3/5/16/17*.
**Figure S10** Association analysis between different haplotypes of *TaCYP78A3/5/16/17* and yield‐related traits of 323 wheat accessions at 16 environmental sites.
**Figure S11** Association analysis of natural variations in *TaCYP78A3/5/16/17‐A* promoters with some yield‐related traits in wheat.
**Figure S12** The expression level of *TaCYP78A3/5/16/17‐A* in spikes of accessions with different haplotypes *of TaCYP78A3/5/16/17‐Ap*.
**Figure S13** Comparison of the targeted sequences of sgRNAs in *TaCYP78A3/5/17‐A* and their mutant sequences.
**Figure S14** Effects of *TaCYP78A3/5/16/17‐A* activity on wheat yield‐related traits.
**Figure S15** Venn diagram of differentially expressed genes among *TaCYP78A3/5/17‐A* overexpressing plant.
**Figure S16** Gene Ontology (GO) and Kyoto Encyclopedia of Genes and Genomes (KEGG) enrichment analysis of common differentially expressed genes (CDEGs) between wild type and *TaCYP78A3/5/16/17‐A* overexpressing plants.
**Figure S17** Cytological observation of organs from *Hap‐H* and *Hap‐G* accessions.
**Figure S18** Correlation analysis of organ size with cell number or cell size.
**Figure S19** Aggregation effect of four favorable haplotypes of *TaCYP78A3/5/16/17‐Ap* on yield and related traits at different environments.
**Figure S20** Aggregation of favorable haplotypes of *TaCYP78A3/5/16/17‐Ap* affected yield‐related traits.
**Figure S21** Compare the phenotypic variance explanation (PVE) and the expression levels of *TaCYP78A3/5/16/17‐A* in different organs.
**Figure S22** Comparison of the transcripts accumulation of *TaCYP78A3/5/16/17‐A* in each organ from *Hap‐H* and *Hap‐G* accessions.
**Figure S23** Frequency of favorable haplotype combinations of *TaCYP78A3/5/16/17‐Ap* in the past 60 years of wheat breeding in China.
**Figure S24** Variation trends of yield‐related traits during wheat breeding in China.

**Table S1** Conserved domains analysis of CYP78A family in plants.
**Table S2** Molecular diversity analysis of *TaCYP78A* family in *Triticum* L.
**Table S2‐1** The information of different wheat populations in the database.
**Table S2‐2** Tajima's D test of the promoter region of *TaCYP78A* family in Chinese Cultivars.
**Table S3** Haplotype analyses of *TaCYP78A3/5/16/17‐Ap* in *Triticum*.
**Table S3‐1** SNP information of haplotypes of *TaCYP78A3/5/16/17‐Ap* in *Triticum*.
**Table S3‐2** Haplotype information of *TaCYP78A3/5/16/17‐Ap* for accessions in *Triticum*.
**Table S3‐3** The number of haplotypes of *TaCYP78A3/5/16/17‐Ap* in *Triticum*.
**Table S4** Cis‐regulatory elements of haplotypes.
**Table S5** Haplotype information of 323 wheat accessions.
**Table S6** Common differentially expressed genes (CDEGs) of transcriptome.
**Table S7** Number of favorable haplotypes of *TaCYP78A3/5/16/17‐Ap* in 323 wheat accessions.
**Table S8** Primer information.

**Appendix S1** Supplementary methods.

## Data Availability

The raw data of the RNA sequencing experiments in this study have been submitted to the NCBI under BioProject number PRJNA718479 and PRJNA1097470.
