# Supplementary material for: Pleiotropic phenotypic effects of the TaCYP78A family on multiple yield‐related traits in wheat
Source: Plant Biotechnol J. 2024 May 23;22(10):2694–708. doi: 10.1111/pbi.14385 (PMC11536447; doi:10.1111/pbi.14385)
Supplement: Supplementary file 1 — Figure S1 Analysis of conserved domains of CYP78A family in plants. Figure S2 Phylogenetic and genetic diversity analysis of CYP78A family in plants. Figure S3 Changes of nucleotide polymorphisms of TaCYP78A3/5/16/17 during wheat polyploidy and domestication. Figure S4 Changes of the π value of TaCYP78A3/5/16/17 during the domestication in Non‐Chinese common wheat. Figure S5 Evolutionary analysis of different haplotypes of TaCYP78A3/5/16/17‐Ap in wheat. Figure S6 Evolutionary history and distribution of the haplotypes of TaCYP78A3/5/16/17‐Ap. Figure S7 Distribution of different haplotypes of TaCYP78A3/5/16/17‐Ap during wheat domestication. Figure S8 Expression profiles of TaCYP78A3/5/16/17 in major organs of wheat. Figure S9 Known QTLs related to yield‐related traits in the upstream and downstream of TaCYP78A3/5/16/17. Figure S10 Association analysis between different haplotypes of TaCYP78A3/5/16/17 and yield‐related traits of 323 wheat accessions at 16 environmental sites. Figure S11 Association analysis of natural variations in TaCYP78A3/5/16/17‐A promoters with some yield‐related traits in wheat. Figure S12 The expression level of TaCYP78A3/5/16/17‐A in spikes of accessions with different haplotypes of TaCYP78A3/5/16/17‐Ap. Figure S13 Comparison of the targeted sequences of sgRNAs in TaCYP78A3/5/17‐A and their mutant sequences. Figure S14 Effects of TaCYP78A3/5/16/17‐A activity on wheat yield‐related traits. Figure S15 Venn diagram of differentially expressed genes among TaCYP78A3/5/17‐A overexpressing plant. Figure S16 Gene Ontology (GO) and Kyoto Encyclopedia of Genes and Genomes (KEGG) enrichment analysis of common differentially expressed genes (CDEGs) between wild type and TaCYP78A3/5/16/17‐A overexpressing plants. Figure S17 Cytological observation of organs from Hap‐H and Hap‐G accessions. Figure S18 Correlation analysis of organ size with cell number or cell size. Figure S19 Aggregation effect of four favorable haplotypes of TaCYP78A3/5/16/17‐Ap on yield [file PBI-22-2694-s003.docx]

**Pleiotropic Phenotypic Effects of the *TaCYP78A* Family on Multiple Yield-Related Traits in Wheat**

Meng Ma^1,2#*^, Linnan Wu^1#^, Mengyao Li^1#^, Long Li^3#^, Lijian Guo^1,4^, Deyan Ka^1^, Tianxing Zhang^5^, Mengdie Zhou^1^, Baowei Wu^1^, Haixia Peng^6^, Zhaoxin Hu^7^, Xiangli Liu^1^, Ruilian Jing^3*^, Huixian Zhao^1,2*^

^1^ College of Life Sciences, Northwest A & F University, Yangling, Shaanxi, 712100, China;

^2^ National Key Laboratory of Crop Improvement for Stress Tolerance and Production, Northwest A & F University, Yangling, Shaanxi, 712100, China

^3^ National Key Facility for Crop Gene Resources and Genetic Improvement / Institute of Crop Science, Chinese Academy of Agricultural Sciences, Beijing, 100081, China

^4^ State Key Laboratory of Aridland Crop Science, Gansu Agricultural University, Lanzhou, 730070, China

^5^ College of Agronomy, Northwest A&F University, Yangling, Shaanxi, 712100, China

^6^ College of Landscape Architecture and Art, Northwest A&F University, Yangling, Shaanxi, 712100, China

^7^ Department of electrical and computer engineering, University of California San Diego, La Jolla, CA 92093, USA

^#^ These authors contributed equally.

*Corresponding: Huixian Zhao ([hxzhao212@nwafu.edu.cn](mailto:hxzhao212@nwafu.edu.cn)); Ruilian Jing, ([jingruilian@caas.cn](mailto:jingruilian@caas.cn)); Meng Ma ([mengma5@nwafu.edu.cn](mailto:mengma5@nwafu.edu.cn)).


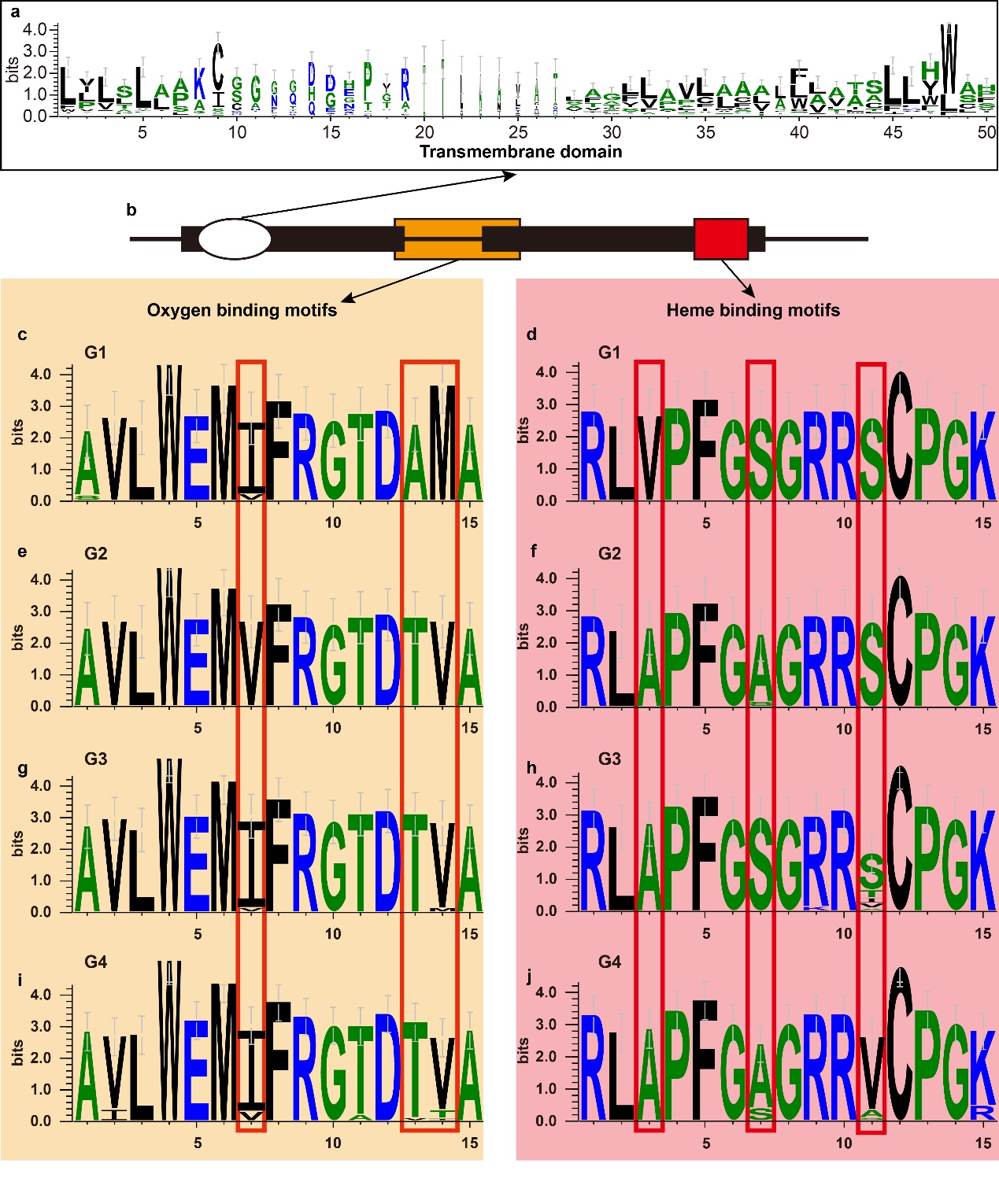


**Supplementary Figure 1. Analysis of conserved domains of *CYP78A* family in plants.**

The analysis of conserved domains was performed as previously described (Chapple, 1998).


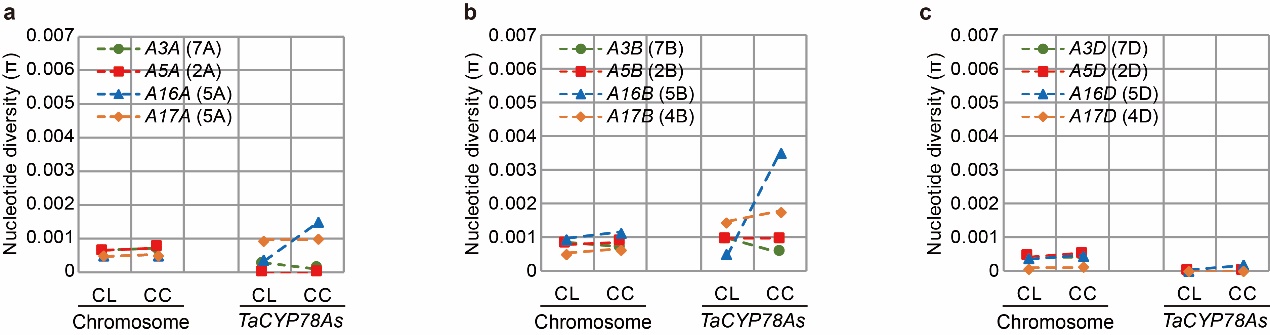


**Supplementary Figure 2. Phylogenetic and genetic diversity analysis of *CYP78A* family in plants.**

(a-c) Changes in the π value of the *TaCYP78A3/5/16/17-A/B/D* coding region during the domestication of common wheat in China. *TaCYP78A3/5/16/17-A/B/D* named as *A3/5/16/17A/B/D* for simplicity; The number and letters within brackets indicated the chromosome number of *TaCYP78A3/5/16/17*, respectively. Chromosome: average π value of each chromosome where the *TaCYP78A3/5/16/17* located in; CL: Chinese Landraces; CC: Chinese Cultivars.


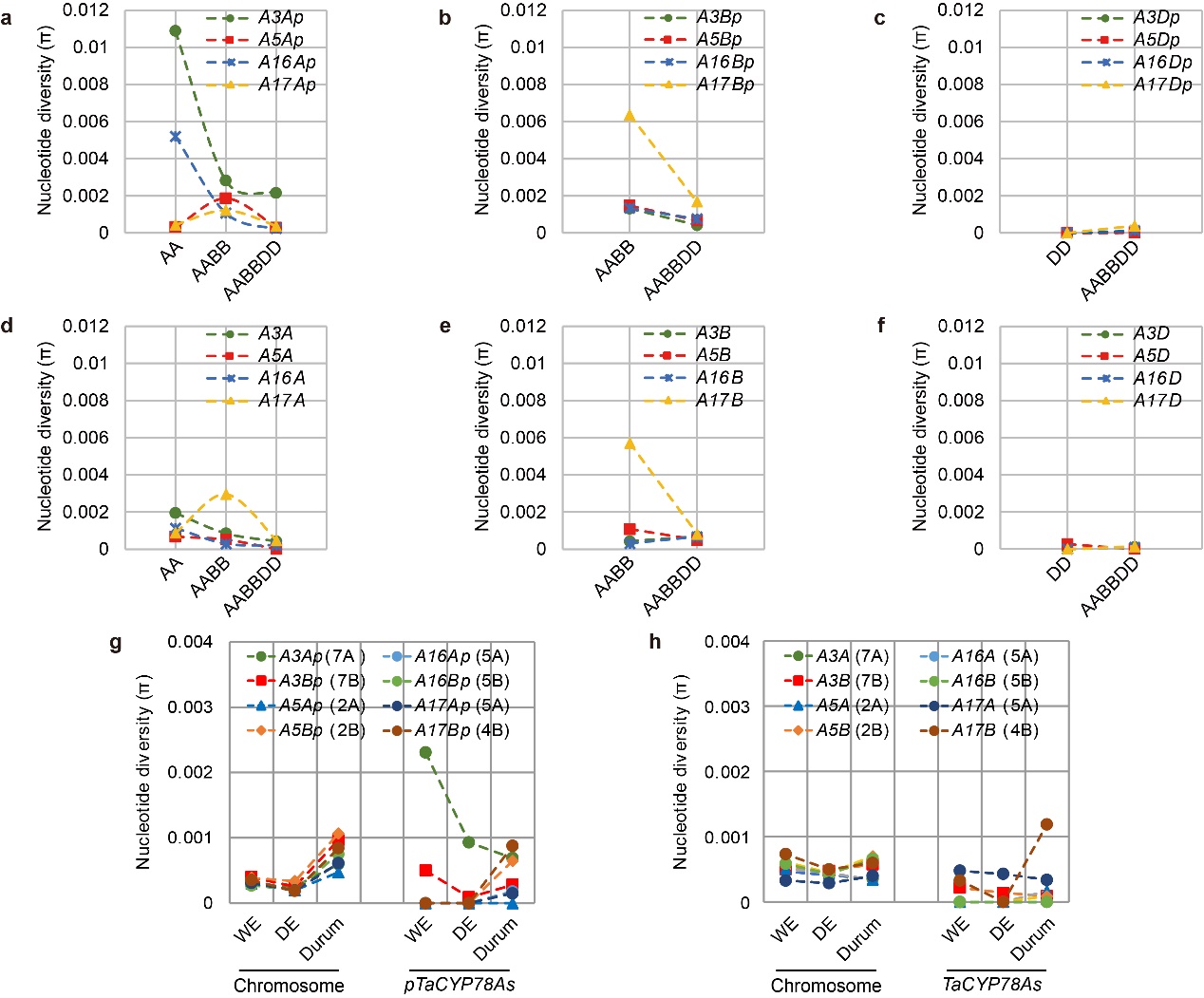


**Supplementary Figure 3. Changes of nucleotide polymorphisms of *TaCYP78A3/5/16/17* during wheat polyploidy and domestication**.

(a-c) Changes in nucleotide polymorphisms in the promoter regions of *TaCYP78A3/5/16/17-A* (a), *TaCYP78A3/5/16/17-B* (b), and *TaCYP78A3/5/16/17-D* (c) during wheat polyploidy. (d-f) Changes of nucleotide polymorphisms in coding regions of *TaCYP78A3/5/16/17-A* (d), *TaCYP78A3/5/16/17-B* (e), and *TaCYP78A3/5/16/17-D* (f) during wheat polyploidy. AA: diploid wheat population with genome AA; DD: diploid wheat population with genome DD; AABB: tetraploid population wheat with genome AABB; AABBDD: hexaploid wheat with genome AABBDD. (g) Changes in the π value of the promoter region of *TaCYP78A3/5/16/17-A/B/D* and its chromosome during wheat domestication. (h) Changes in the π value of the coding region of *TaCYP78A3/5/16/17-A/B/D* and its chromosome during wheat domestication. Chromosome: average π value of the chromosome where the *TaCYP78A3/5/16/17* located in; WE: Wild Emmer; DE: Domesticated Emmer. *TaCYP78A3/5/16/17-A/B/D* named as *A3/5/16/17A/B/D* for simplicity; The promoters of *TaCYP78A3/5/16/17-A/B/D* named as *A3/5/16/17A/B/Dp* for simplicity; The number and letters within brackets indicated the chromosome number of *TaCYP78A3/5/16/17*, respectively.


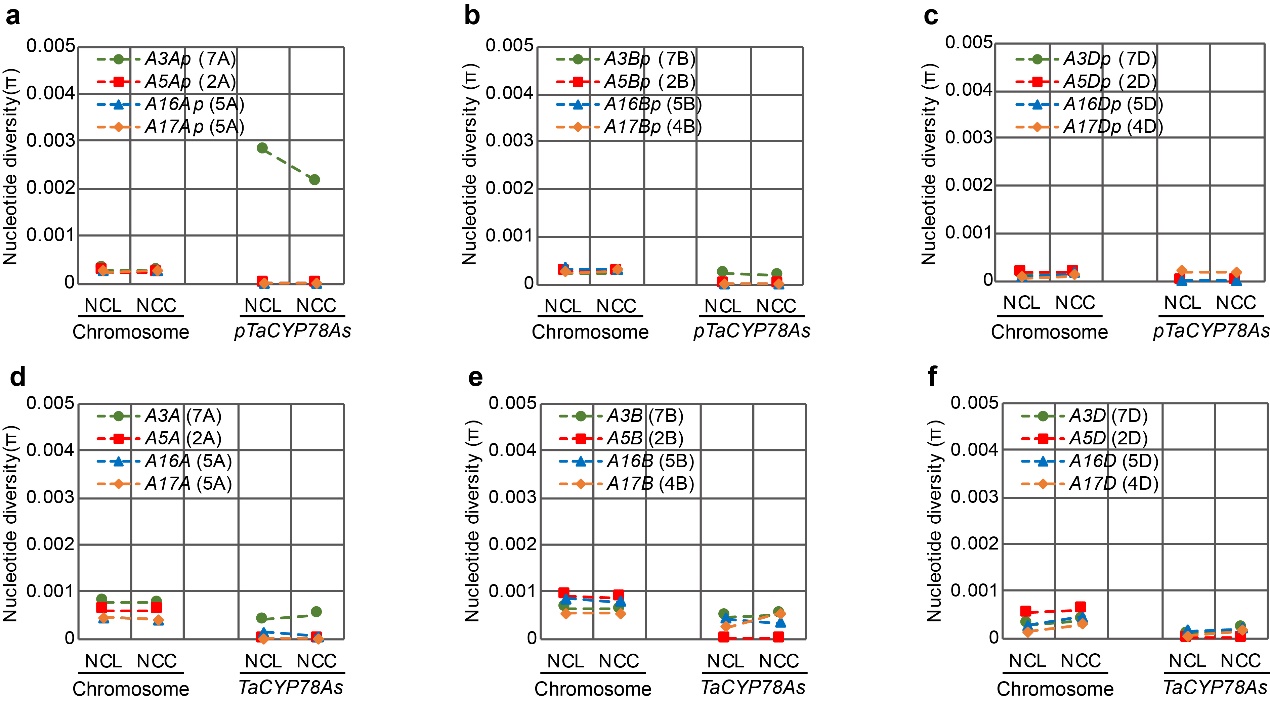


**Supplementary Figure 4. Changes of the π value of *TaCYP78A3/5/16/17* during the domestication in Non-Chinese common wheat.**

(a-c) Changes in the π value of the *TaCYP78A3/5/16/17* promoter region and its chromosome during breeding of Non-Chinese common wheat. (d-f) Changes in the π value of the *TaCYP78A3/5/16/17* coding region and its chromosome during the breeding of Non-Chinese common wheat. Chromosome: average π value of chromosome; NCL: Non-Chinese Landraces; NCC: Non-Chinese Cultivars. *TaCYP78A3/5/16/17-A/B/D* named as *A3/5/16/17A/B/D* for simplicity; The promoters of *TaCYP78A3/5/16/17-A/B/D* named as *A3/5/16/17A/B/Dp* for simplicity; The number and letters within brackets indicated the chromosome number of *TaCYP78A3/5/16/17*, respectively.


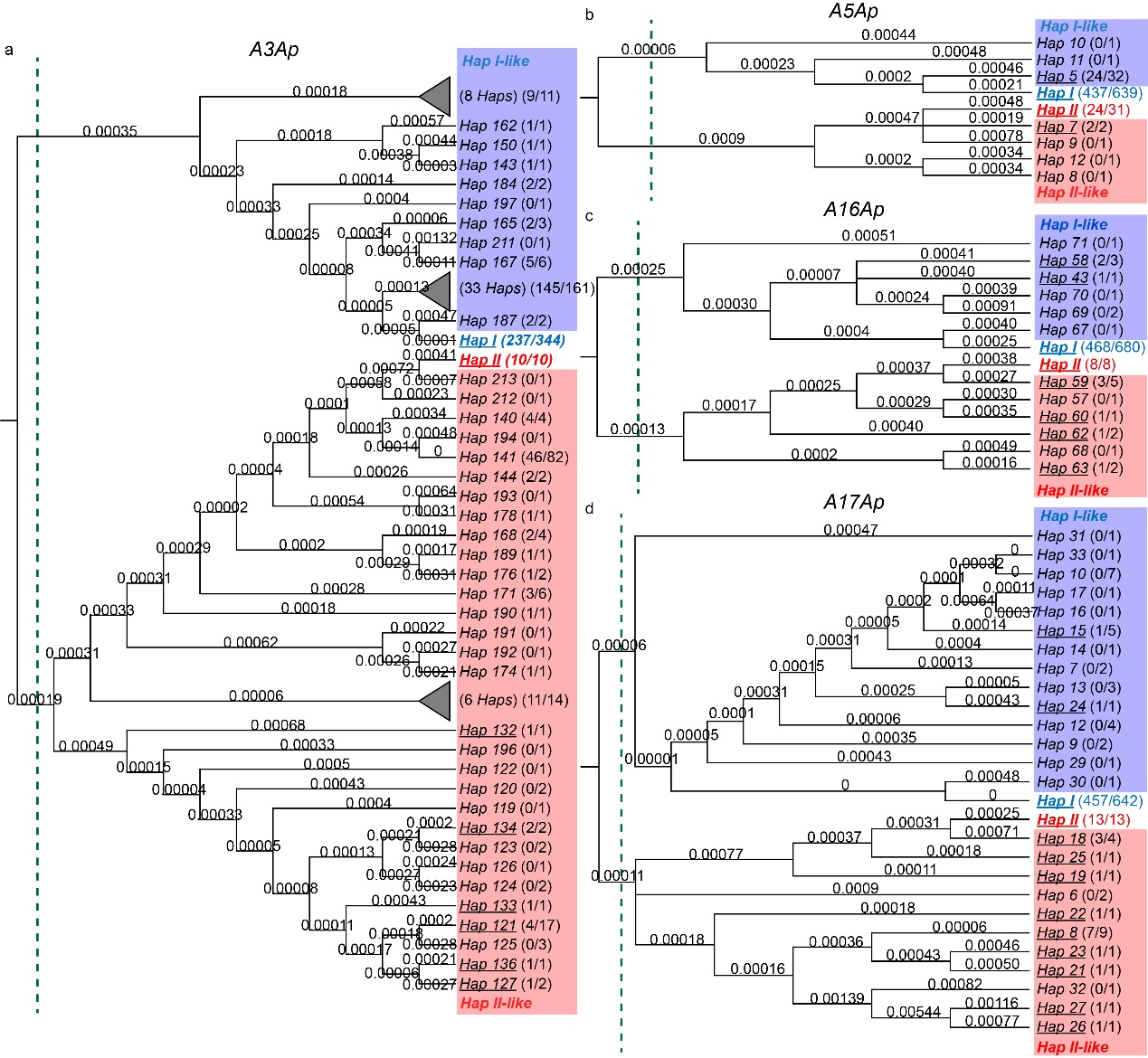


**Supplementary Figure 5. Evolutionary analysis of different haplotypes of *TaCYP78A3/5/16/17-Ap* in** **wheat*.***

(a-d): Evolutionary tree of *TaCYP78A3-Ap* (a), *TaCYP78A5-Ap* (b), *TaCYP78A16-Ap* (c) and *TaCYP78A17-Ap* (d) haplotypes, respectively, in cultivars (n=489) and wheat accessions (n=791) presented in Supplementary Table 2-3. The numbers on the branches indicate genetic distances; The number in brackets indicate the number of accessions with this haplotype in cultivars/wheat accessions.


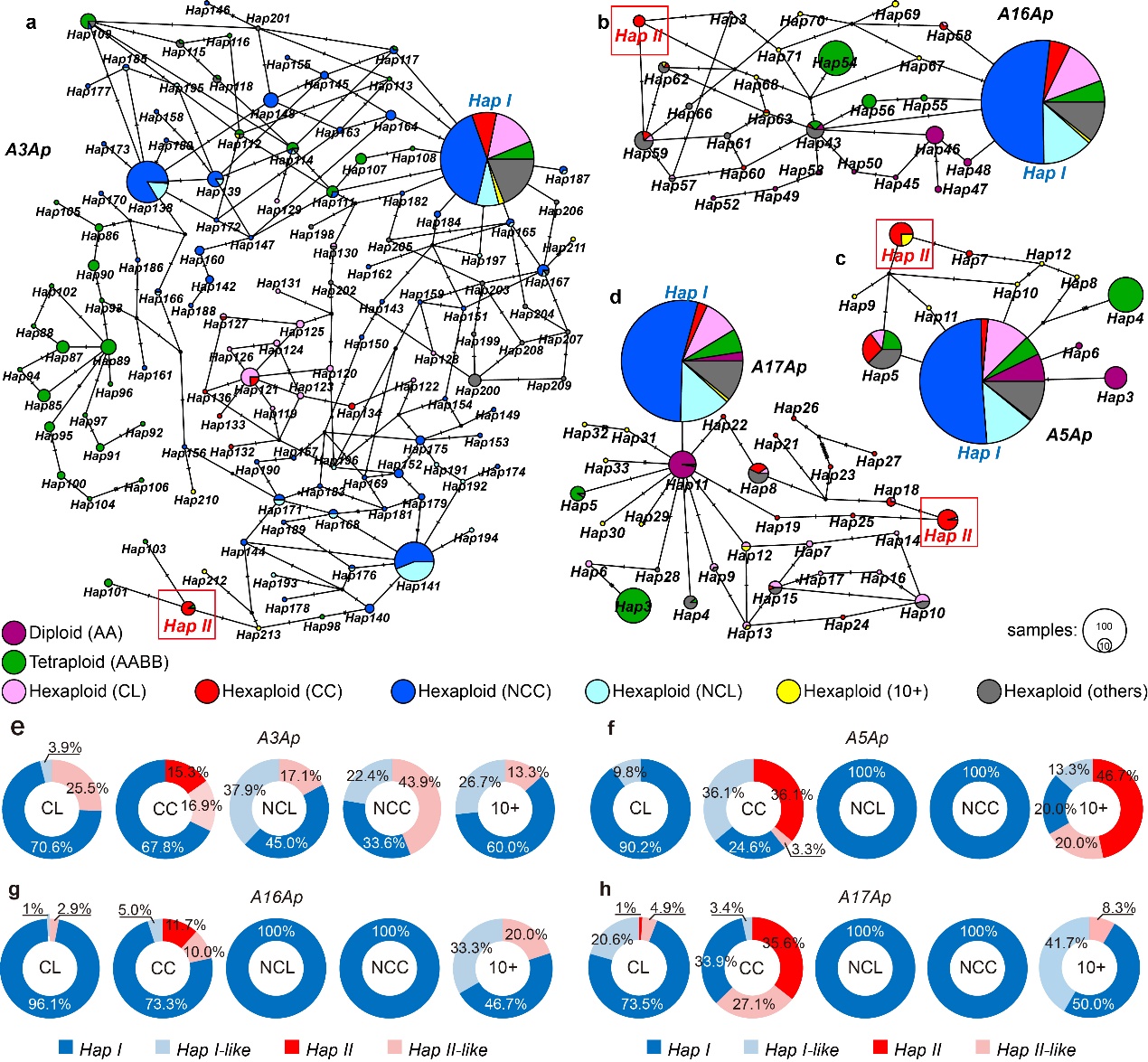


**Supplementary Figure 6. Evolutionary history and distribution of the haplotypes of *TaCYP78A3/5/16/17-Ap*.**

(a-b) Partial haplotype networks of *TaCYP78A3-Ap* (a) and *TaCYP78A16-Ap* (b). (c-d) Haplotype networks of *TaCYP78A5-Ap* (c) and *TaCYP78A17-Ap* (d). *TaCYP78A3/5/16/17-A* promoters named as *A3/5/16/17Ap*s for simplicity*,* respectively. Each circle represents a haplotype, and the black solid points represent the putative intermediate haplotypes. The short black lines on the line connecting the haplotypes represent the relative distances of the two haplotypes it connects, with more dashes representing a greater relative distance. (e-h) Distribution frequencies of *TaCYP78A3-Ap-HapI/II* (e), *TaCYP78A5-Ap-HapI/II* (f), *TaCYP78A16-Ap-HapI/II* (g) and *TaCYP78A17-Ap-HapI/II* (h) in different wheat populations. CL: Chinese Landraces; CC: Chinese Cultivars; NCL: Non-Chinese Landraces; NCC: Non-Chinese Cultivars; 10+: 10+ Wheat Genomes lines. CC, CL, NCC, NCL, and 10+ contain 43, 102, 422, 107, and 15 wheat accessions, respectively.

**
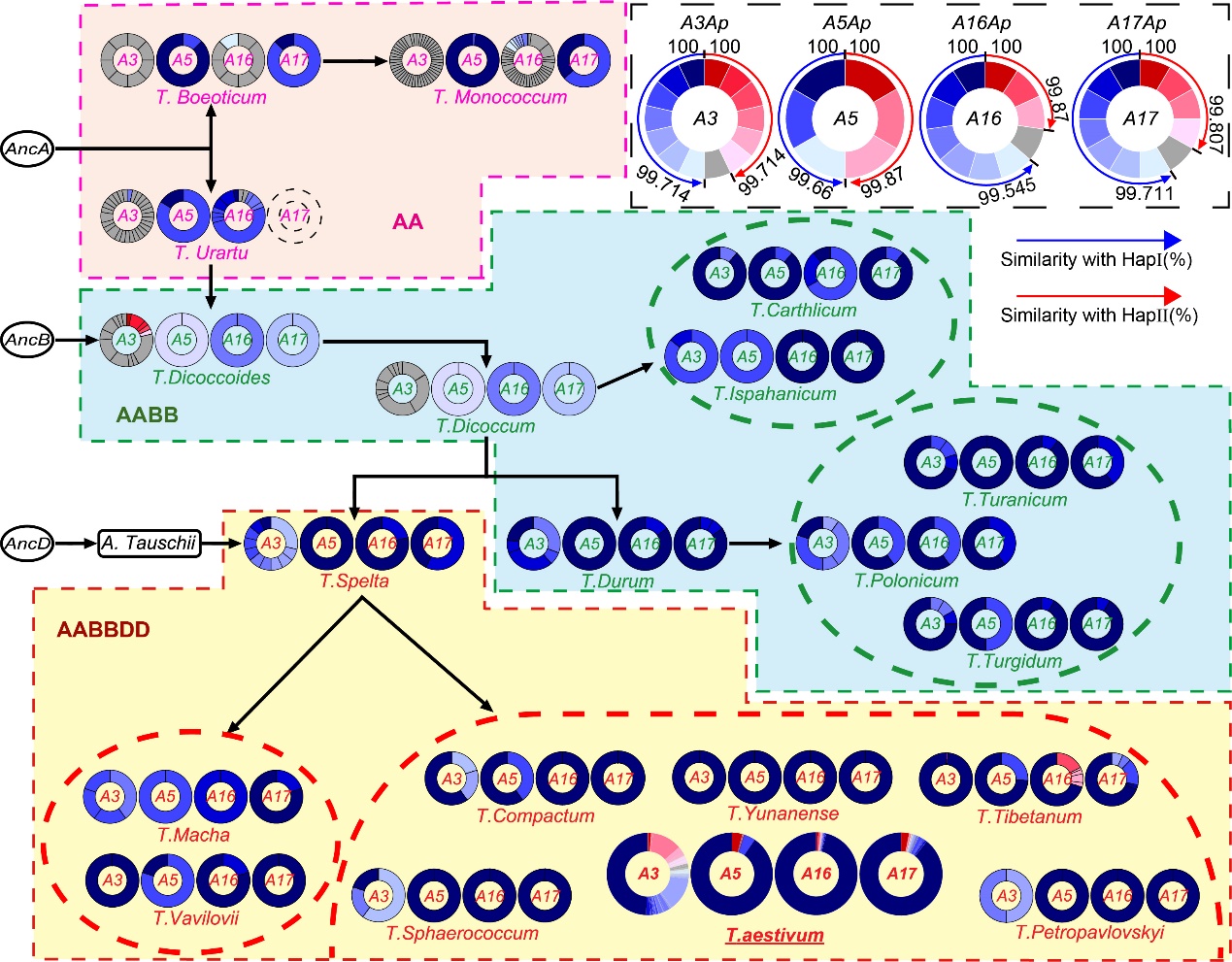
**

**Supplementary Figure 7. Distribution of different haplotypes of *TaCYP78A3/5/16/17-Ap* during wheat domestication.**

Each circle represents a haplotype of *TaCYP78A3/5/16/17-Ap* (The haplotypes of *TaCYP78A3/5/16/17-Ap* are named as *A3/5/16/17* for simplicity*,* respectively). Colors within the circle from dark blue to light blue or from dark red to light red respectively indicate a decrease in sequence similarity between different genotypes compared to *TaCYP78A3/5/16/17-Ap-Hap I* or *-Hap II*, respectively, and the proportion of colors represents the proportion of different genotypes in all haplotypes of *TaCYP78A3/5/16/17-Ap*. Arrows mark the direction of wheat domestication.


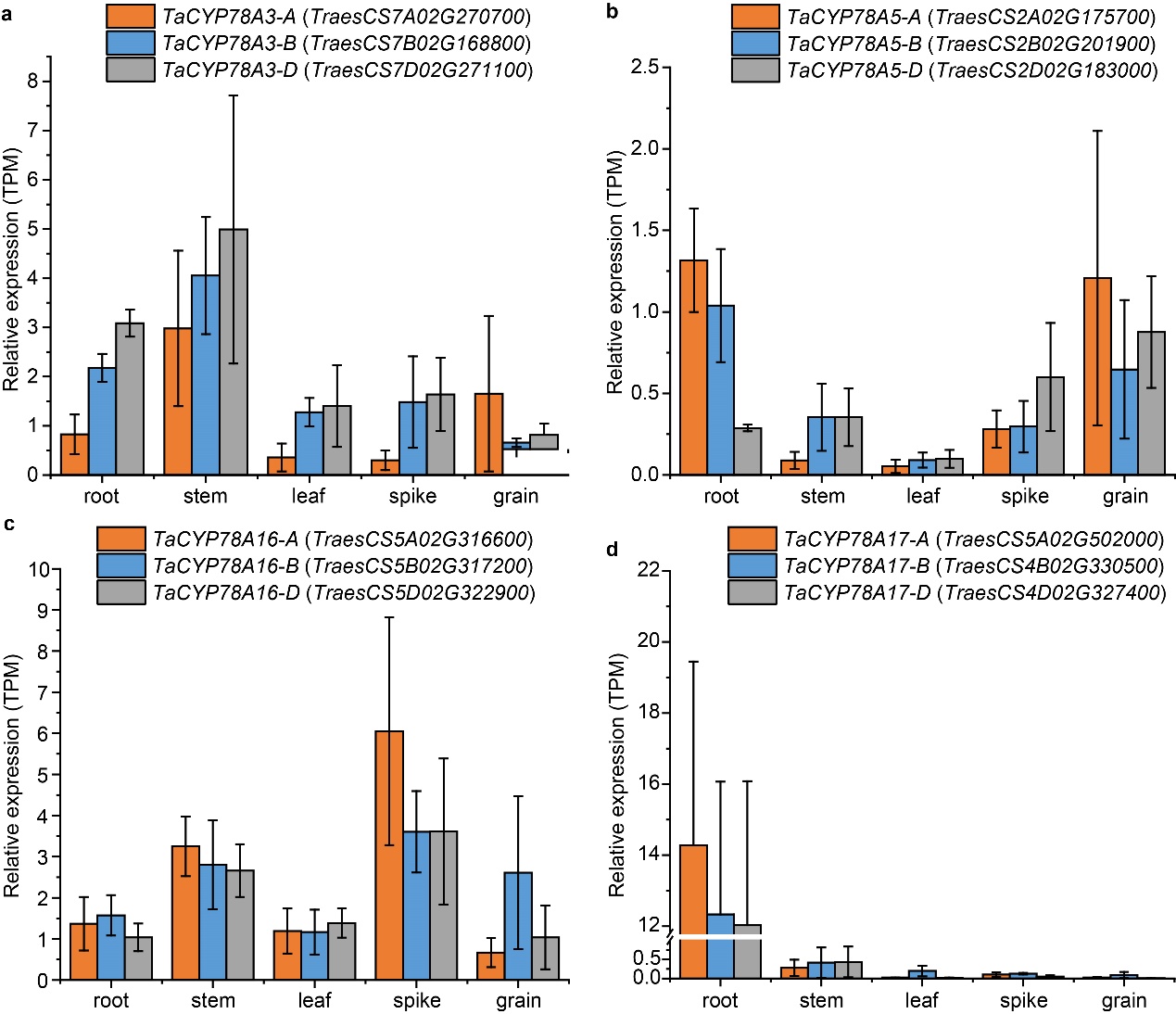


**Supplementary Figure 8. Expression profiles of *TaCYP78A3/5/16/17* in major organs of wheat.**

Expression data derived from the transcriptome data of previous study (Chi et al., 2019).


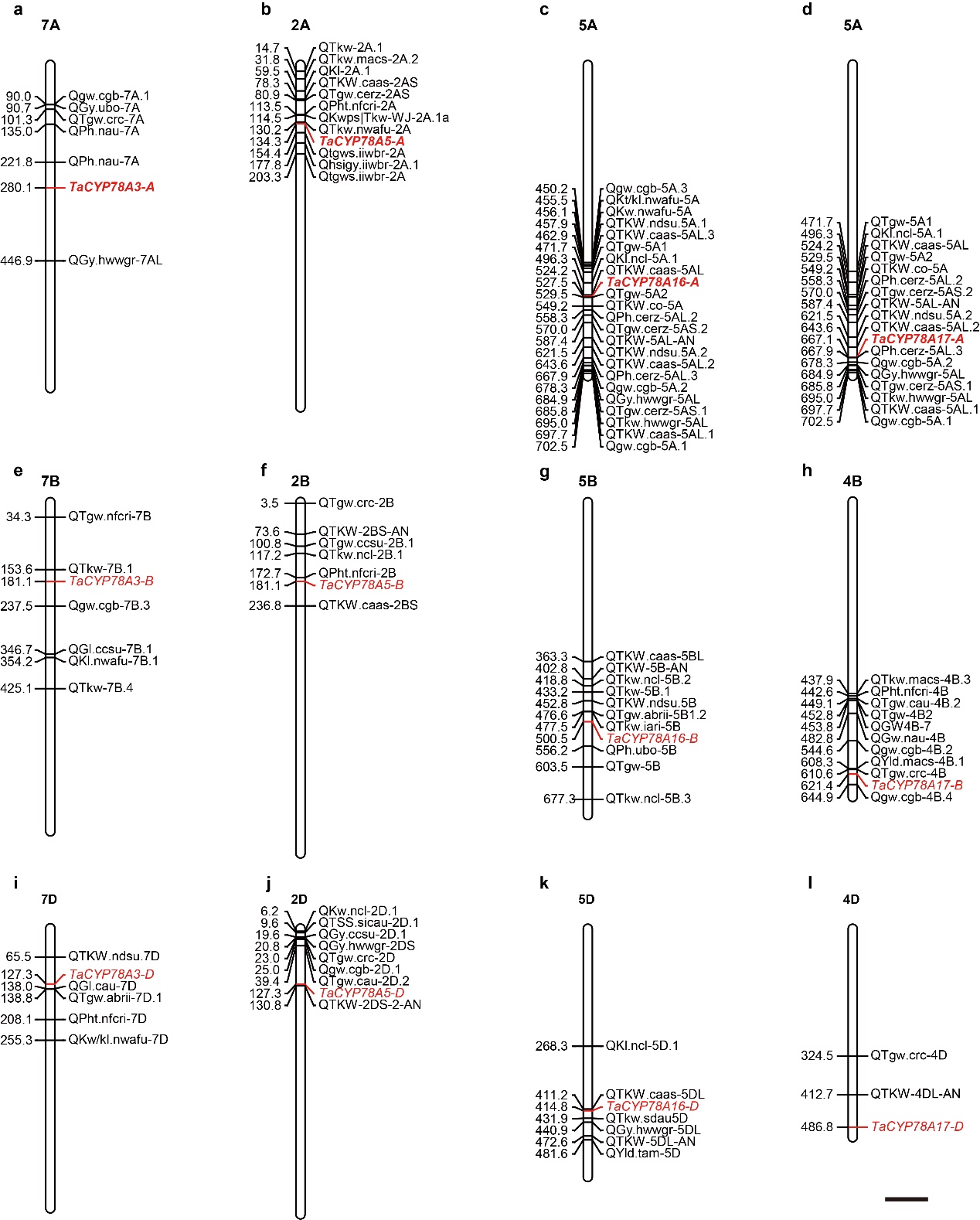


**Supplementary Figure 9. Known QTLs related to yield-related traits in the upstream and downstream of *TaCYP78A3/5/16/17.***


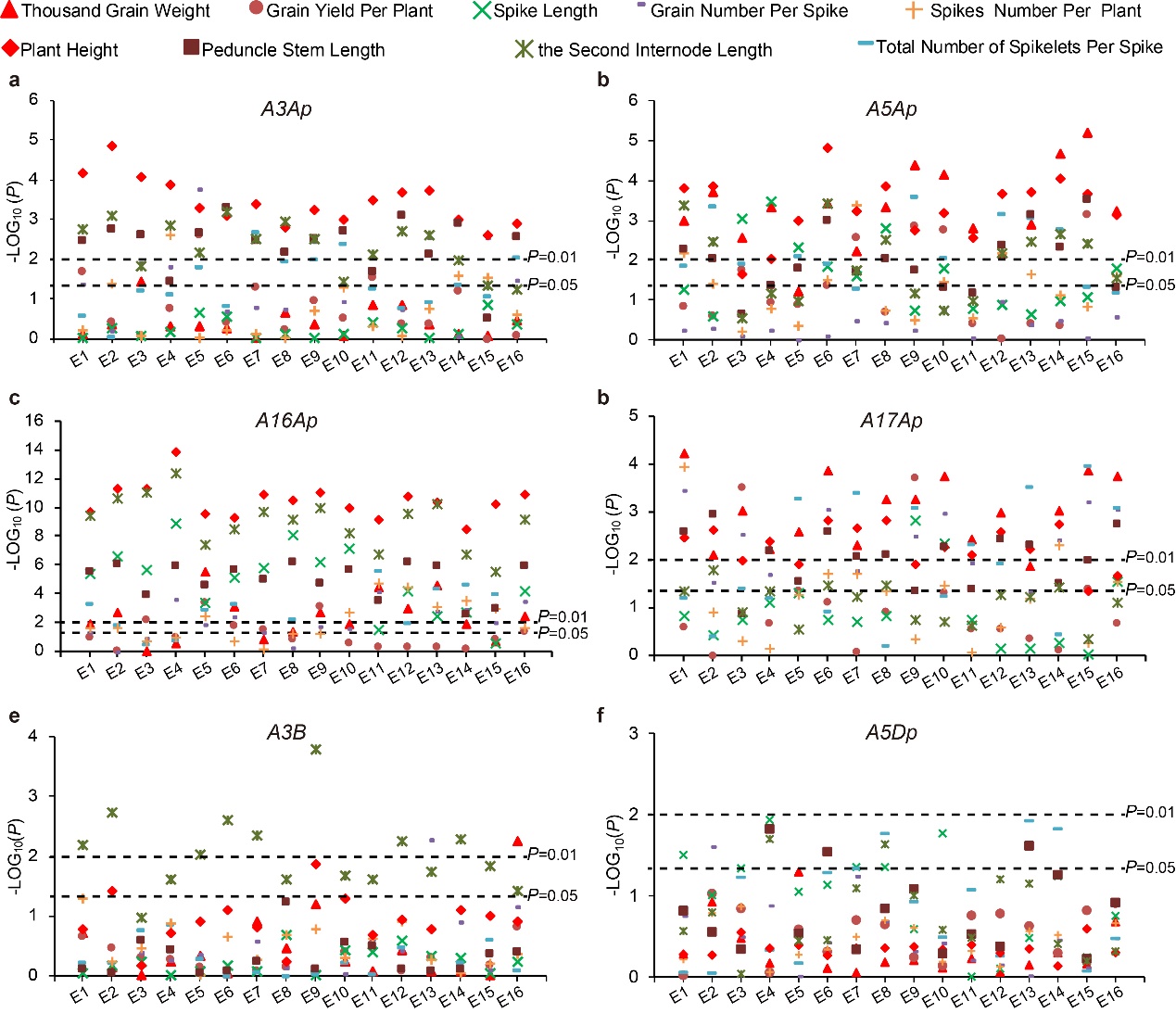


**Supplementary Figure 10. Association analysis between different haplotypes of *TaCYP78A3/5/16/17* and yield-related traits of 323 wheat accessions at 16 environmental sites.**

(a-d): The *P-Value* of the correlation analysis of *TaCYP78A3/5/16/17-A* promoter (*A3/5/16/17Ap*) haplotypes and yield-related traits at 16 environmental points (E1-E16), respectively. (e-f): The *P-Value* of association analysis of different haplotypes of *TaCYP78A3-B* coding region (*A3B*, e) and *TaCYP78A5-D* promoter region (*A5Dp*, f) and yield-related traits at 16 environmental points (E1-E16), respectively. Data of phenotypic were from 323 wheat accessions at 16 environmental sites.


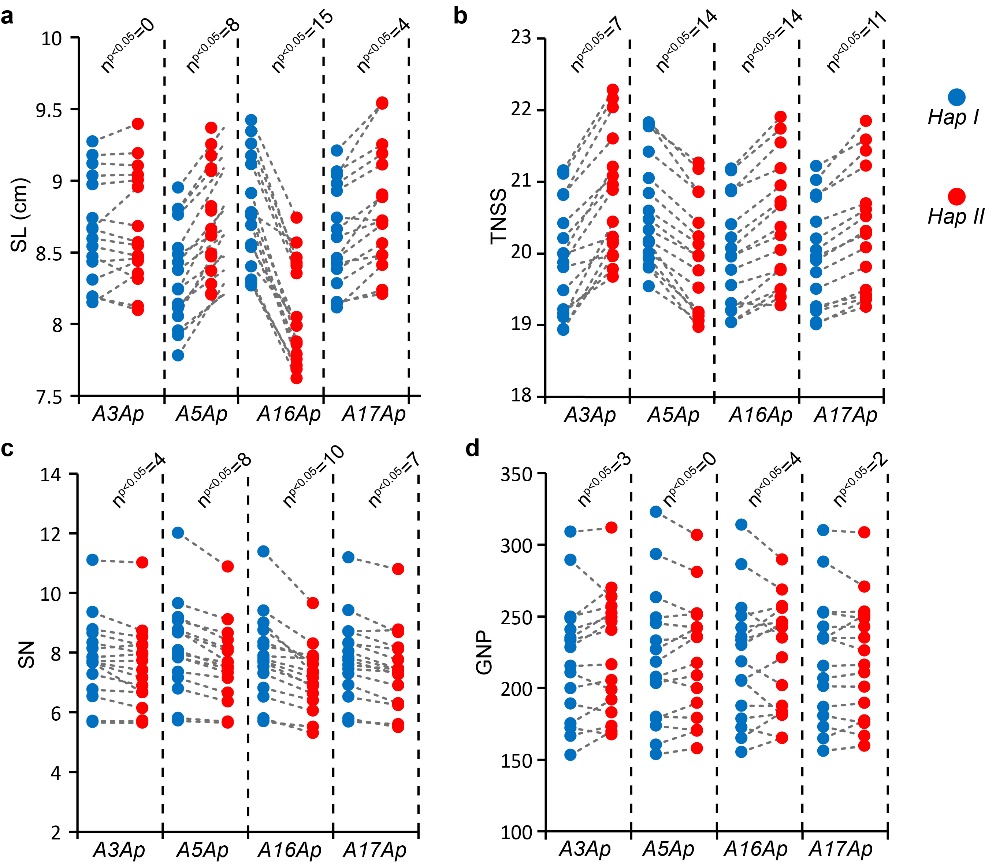


**Supplementary Figure 11. Association analysis of natural variations in *TaCYP78A3/5/16/17-A* promoters with some yield-related traits in wheat.**

(a-d): Statistical analysis of spike length (SL, a), total number of spikelets per spike (TNSS, b), spike number per plant (SNPP, c) and grain number per plant (GNP, d) of wheat accessions with different *TaCYP78A3/5/16/17-Ap* haplotypes at 16 environmental sites. *A3Ap*, *A5Ap*, *A16Ap*, and *A17Ap* represent the promoters of *TaCYP78A3/5/16/17-A*, respectively. *HapI* and *HapII* indicate the two haplotypes of *A3Ap*, *A5Ap*, *A16Ap*, and *A17Ap*. The individual points represent the averages of phenotypic data of a yield-related trait of wheat accessions with different *TaCYP78A3/5/16/17-Ap* haplotypes at one environmental site. “n” represents the numbers of environmental sites where there is significant difference in each yield-related trait between accessions with *HapI* and *HapII* of *A3Ap*, *A5Ap*, *A16A*p, and *A17Ap*. *TaCYP78A3/5/16/17Ap-HapI/II* haplotypes contain 246/77, 58/265, 266/57 and 232/91 accessions, respectively. *P* < 0.05 and *P* < 0.01 by Student’s *t*-test.


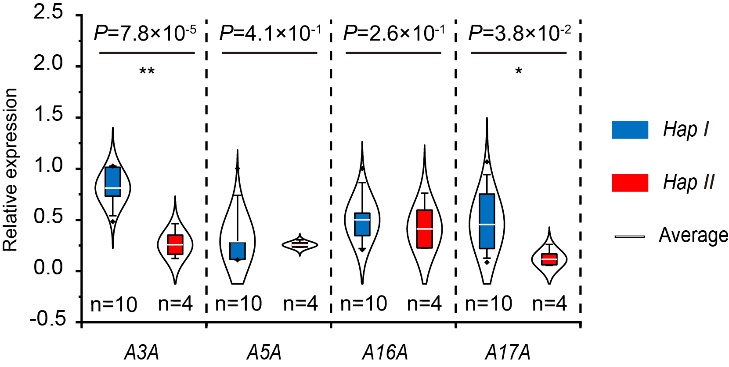


**Supplementary Figure 12. The expression level of *TaCYP78A3/5/16/17-A* in spikes of accessions with different haplotypes *of TaCYP78A3/5/16/17-Ap*.**

The data of the expression level of *TaCYP78A3/5/16/17-A* in spikes represent the average ±SE (Standard Error) of the three biological replicates of "n" wheat accessions (at booting stage). **P* < 0.05, ***P* < 0.01 by Student’s *t*-test. *TaCYP78A3/5/16/17-A* named as *A3/5/16/17A*, respectively, for simplicity.


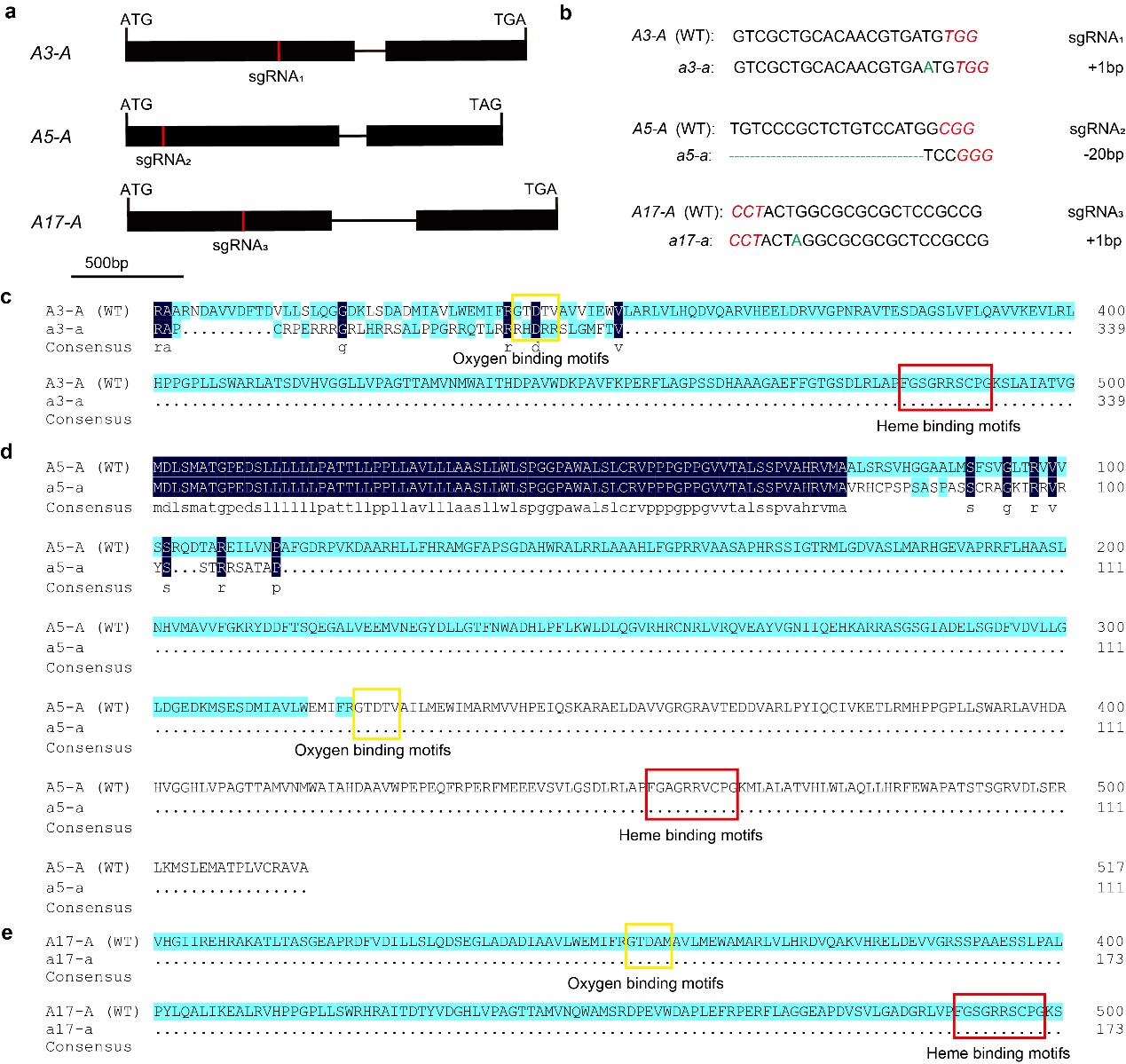


**Supplementary Figure 13. Comparison of the targeted sequences of sgRNAs in *TaCYP78A3/5/17-A* and their mutant sequences.**

(a) Schematic diagrams of *TaCYP78A3/5/17* gene structures and targeted positions of CRISPR-Cas9. Black boxes, exons; Central black line, intron. The red labels indicate the positions of targeted sequences. *A3/5/17-A* represent *TaCYP78A3/5/17-A*, respectively. (b) Display of targeted sequences and flanking sequences of sgRNAs in *A3/5/17-A* and their mutant genes *a3/5/17-a*. The red base is the PAM site, the green base is the insertion base, and the * is the deletion base; the symbols ‘+’ and ‘-’ indicate the base insertion or deletion, respectively. (c-e) Amino acid sequence alignment of TaCYP78A3/5/17 and their mutant proteins. Yellow boxes mark the oxygen-binding domain and red boxes mark the heme-binding domain. Deletion of conserved domains of mutant proteins is shown.


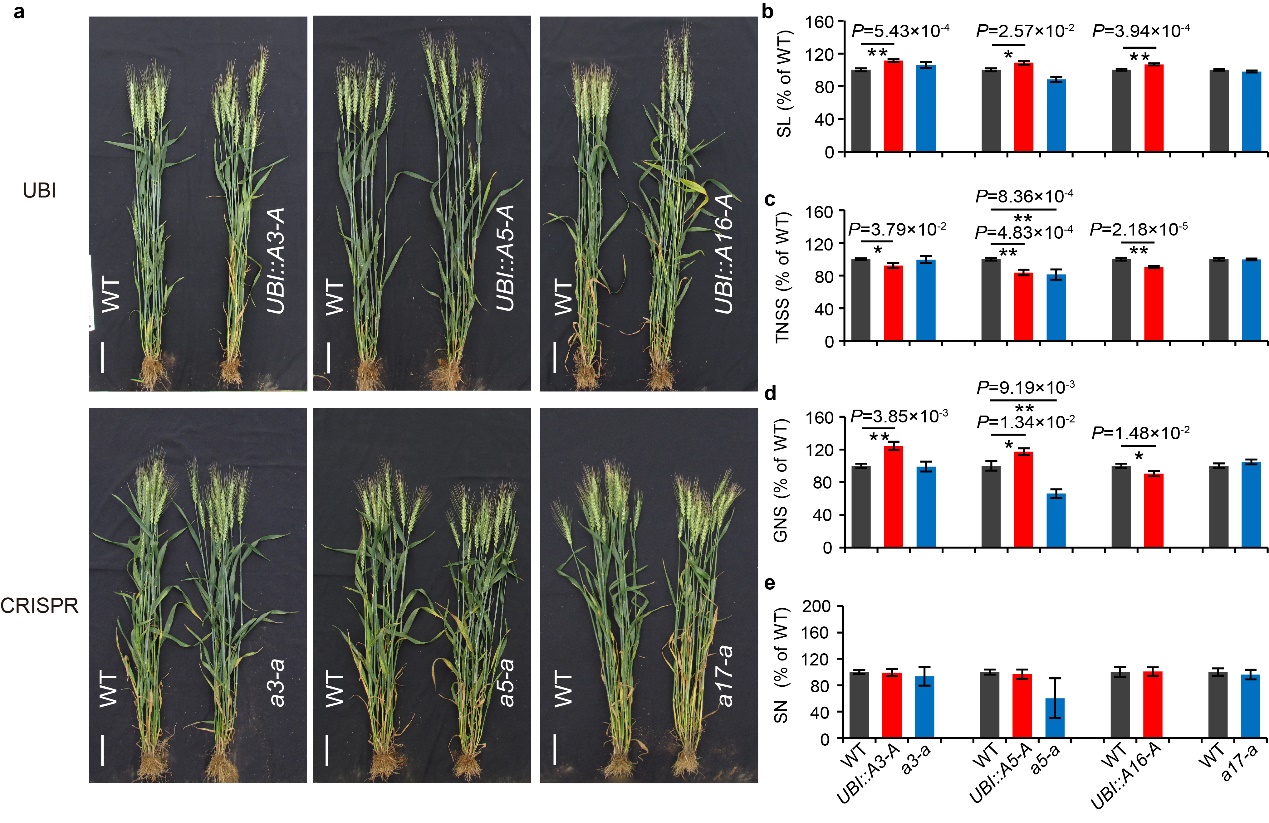


**Supplementary Figure 14. Effects of *TaCYP78A3/5/16/17-A* activity on wheat yield-related traits.**

(a) Comparison of the phenotypes of plants with different genotypes at the grain filling stage. Bars = 10 cm. (b-e) Comparison of spike length (SL, b), total number of spikelet per spike (TNSS, c), grain number per spike (GNS, d) and spike number per plant (SNPP, e) of wild type (WT), *TaCYP78A3/5/16-A* overexpressing (*UBI::A3/5/16-A*) and loss-of-function mutant (*a3/5/17-a*) plants (n>10). Values indicate means ± SE (Standard Error). **P* < 0.05, ***P* < 0.01 by Student’s *t*-test. *TaCYP78A3/5/16/17-A* named as *A3/5/16/17-A* for simplicity, respectively.


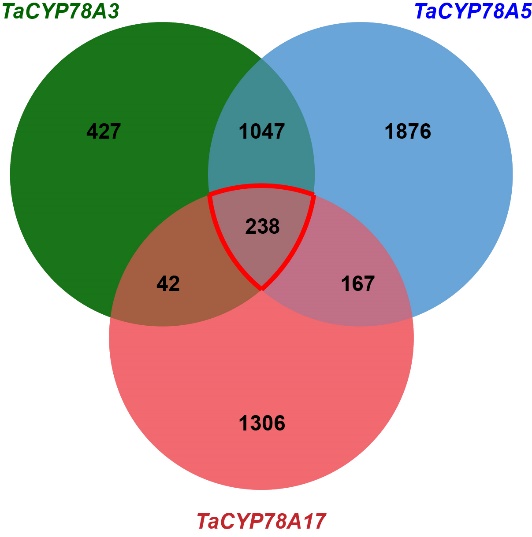


**Supplementary Figure 15. Venn diagram of differentially expressed genes among *TaCYP78A3/5/17-A* overexpressing plant.**


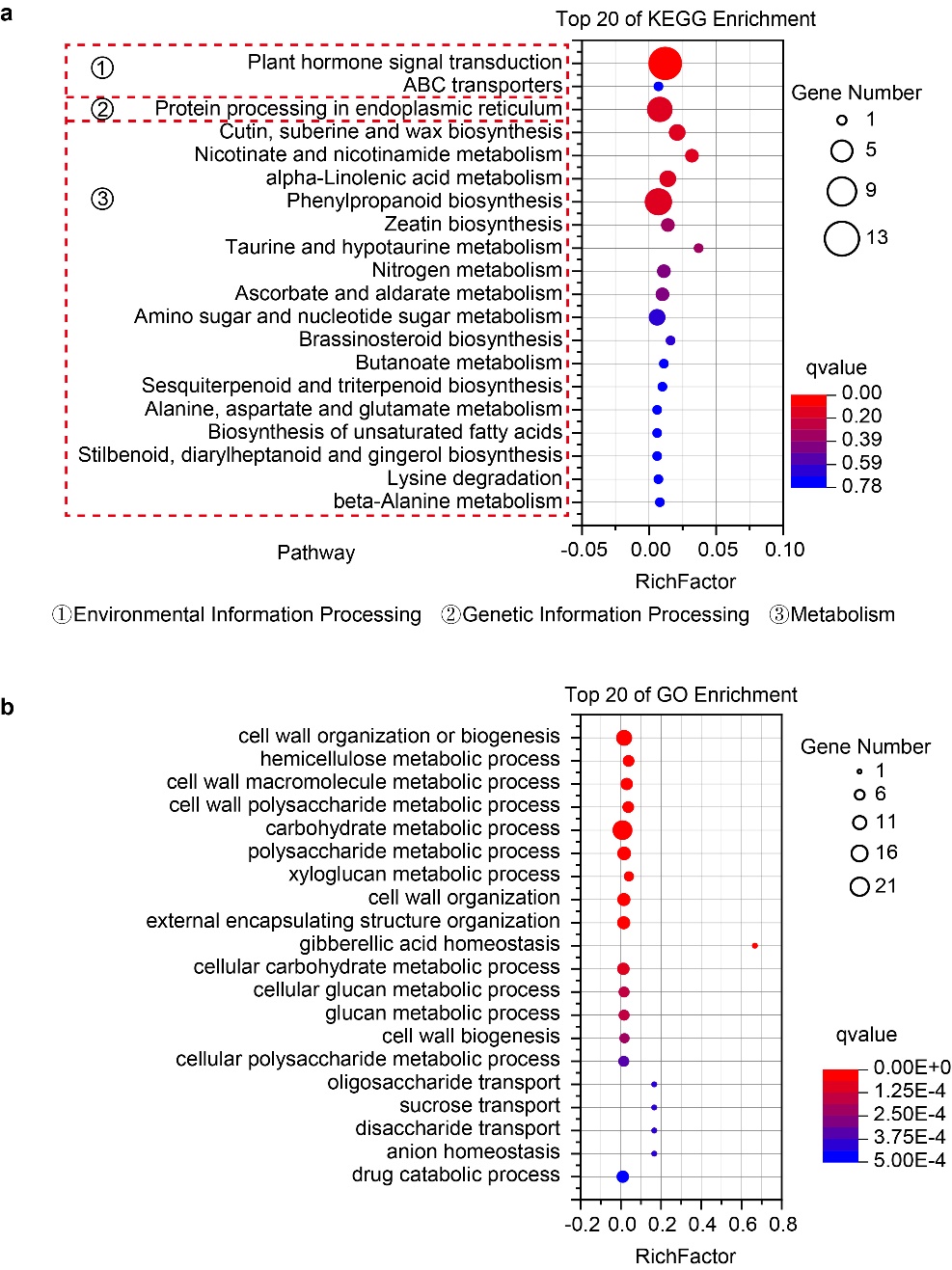


**Supplementary Figure 16. Gene Ontology (GO) and Kyoto Encyclopedia of Genes and Genomes (KEGG) enrichment analysis of common differentially expressed genes (CDEGs) between wild type and *TaCYP78A3/5/16/17-A* overexpressing plants.**

The top 20 significantly enriched pathways using GO categories (a) and KEGG (b) enrichment analysis are shown in the histogram.


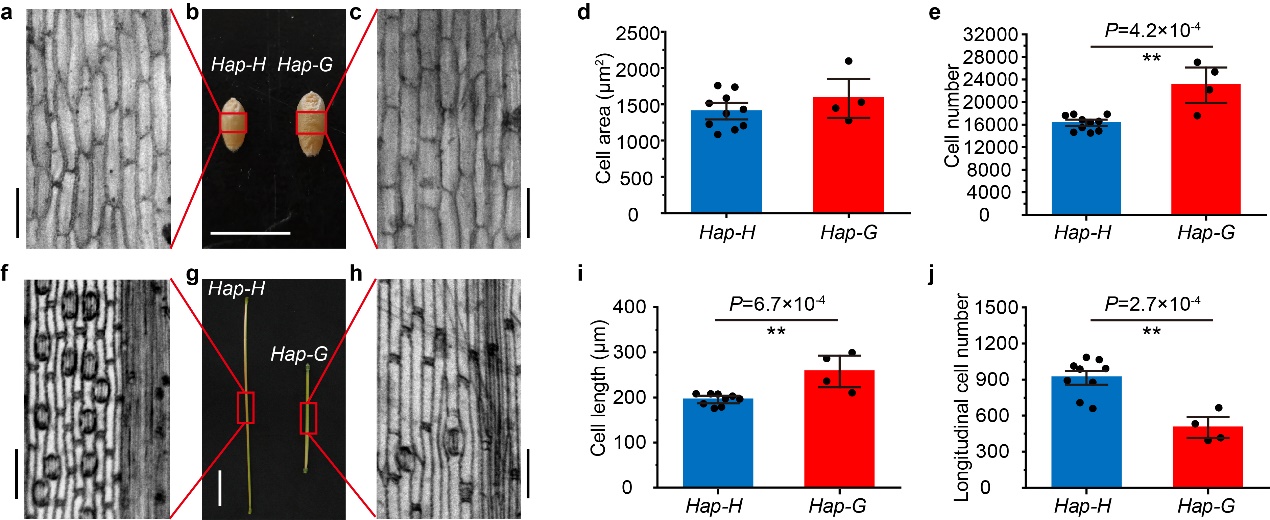


**Supplementary Figure 17. Cytological observation of organs from *Hap-H* and *Hap-G* accessions.**

(a-c) Comparison of mature grains (a) and grain coat cells (a, c) of *Hap-H* (n=10) and *Hap-G* (n=4) accessions. Bars = 100 μm (a, c) and 1 cm (b). (d-e) Statistical analysis of cell area (d) and cell number (e) of (a-c). (f-h) Comparison of stems (g) and their epidermal cells (f, h) of *Hap-H* and *Hap-G* accessions. Bars = 100 μm (f, h) and 5 cm (g). (i-j) Statistical analysis of epidermal cell length (i) and longitudinal cell number (j) of (f-h). *Hap-H*: Genotypes with zero favorable haplotype of *TaCYP78A3/5/16/17-Ap* (*A3/5/16/17Ap-Hap II*); *Hap-G*: Genotypes with four *A3/5/16/17Ap-Hap II*. One point in the bar graph (d-e, i-j) represents one wheat accession, and more than 10 samples × 50 biological replicates were tested for each accession. Values indicate means ± SE. ***P* < 0.01 by Student's *t*-test.


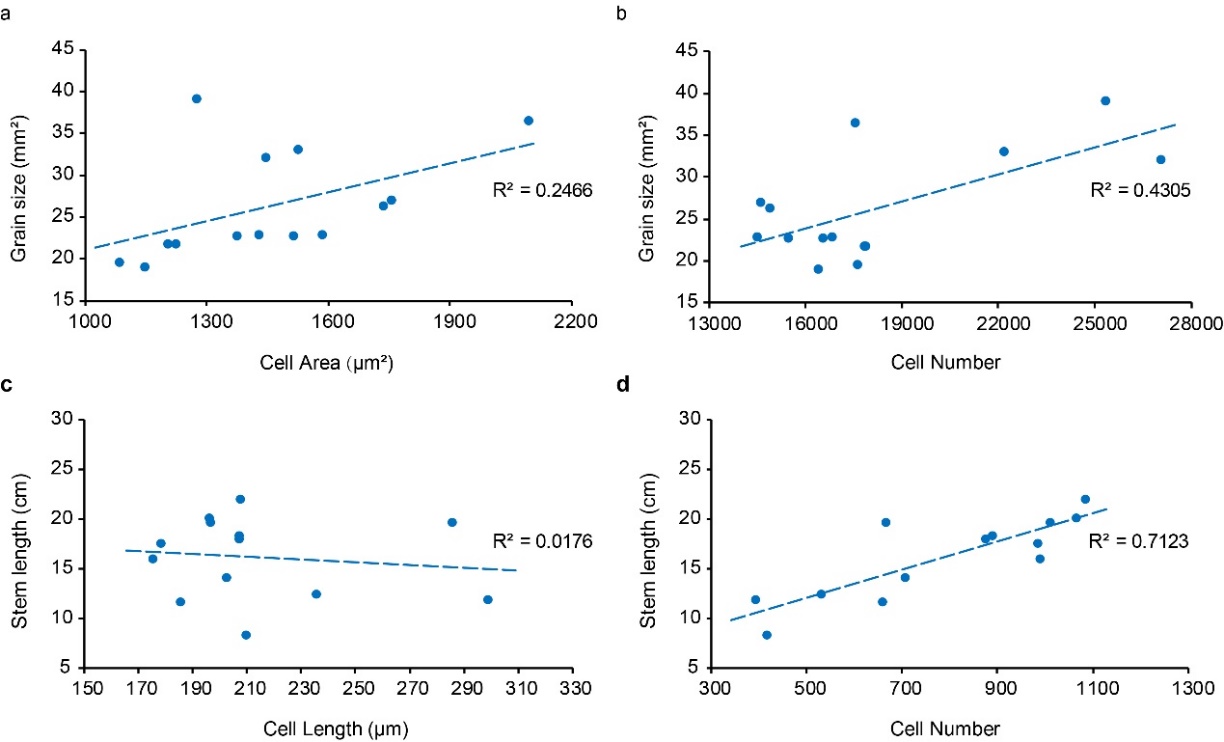


**Supplementary Figure 18. Correlation analysis of organ size with cell number or cell size.**

Epidermal cell characteristics of grains and stems from wheat accessions with *Hap-H* (n=10) or *Hap-G* (n=4) were investigated. *Hap-H*: Genotypes with zero favorable haplotype of *TaCYP78A3/5/16/17-Ap* (*A3/5/16/17Ap-Hap II*); *Hap-G*: Genotypes with four *A3/5/16/17Ap-Hap II*. One point represents the average of phenotypic data for one accession, and more than 10 samples × 50 biological replicates were tested for each accession.


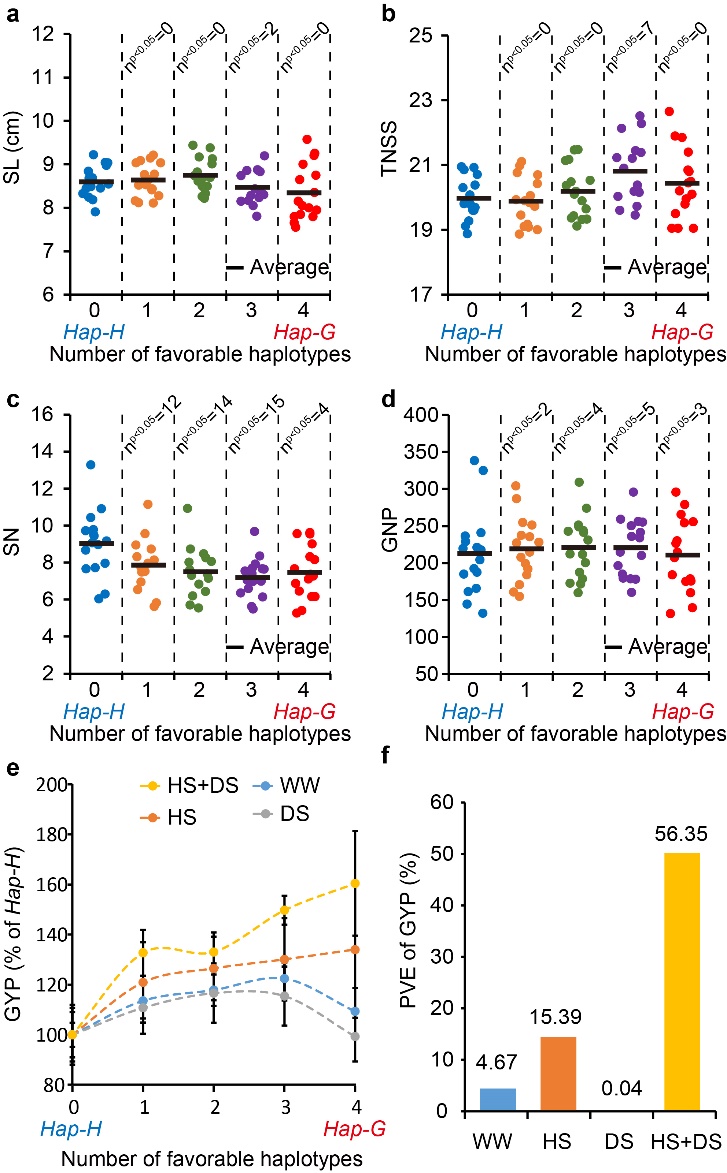


**Supplementary Figure 19. Aggregation effect of four favorable haplotypes of *TaCYP78A3/5/16/17-Ap* on yield and related traits at different environments.**

(a-d) The relationship between the number of favorable haplotypes of *TaCYP78A3/5/16/17-Ap* and spike length (SL, a), total number spikelet per spike (TNSS, b), spikes number per plant (SNPP, c) and grain number per plant (GNP, d). (e) The relationship between the number of favorable haplotypes of *TaCYP78A3/5/16/17-Ap* and grain yield per plant (GYP) at different planting environments. (f) The phenotypic variance explanation (PVE) of the number of favorable haplotypes on GYP at different planting environments. PVE was performed as previously described (Shi et al., 2020). *Hap-H*: Wheat accessions with zero favorable haplotype of *TaCYP78A3/5/16/17-Ap*; *Hap-G*: Wheat accessions with four favorable haplotypes of *TaCYP78A3/5/16/17-Ap*. WW: well water at 5 environmental sites; HS: heat stress at 5 environmental sites; DS: drought stress at 3 environmental sites; DS+HS: drought and heat stress at 3 environmental sites. Phenotypic data were obtained from the 323 wheat accessions planted at 16 environmental sites, and at least ten plants of individual accessions being measured for each trait in each environment. There are 25, 140, 128, 26 and 4 accessions with 0 to 4 favorable haplotypes of *TaCYP78A3/5/16/17-Ap*, respectively. “n” represents the number of environmental sites with significant differences in this YRTs between *Hap-H* and other genotype. *P* < 0.05 and *P* < 0.01 by Student’s *t*-test.


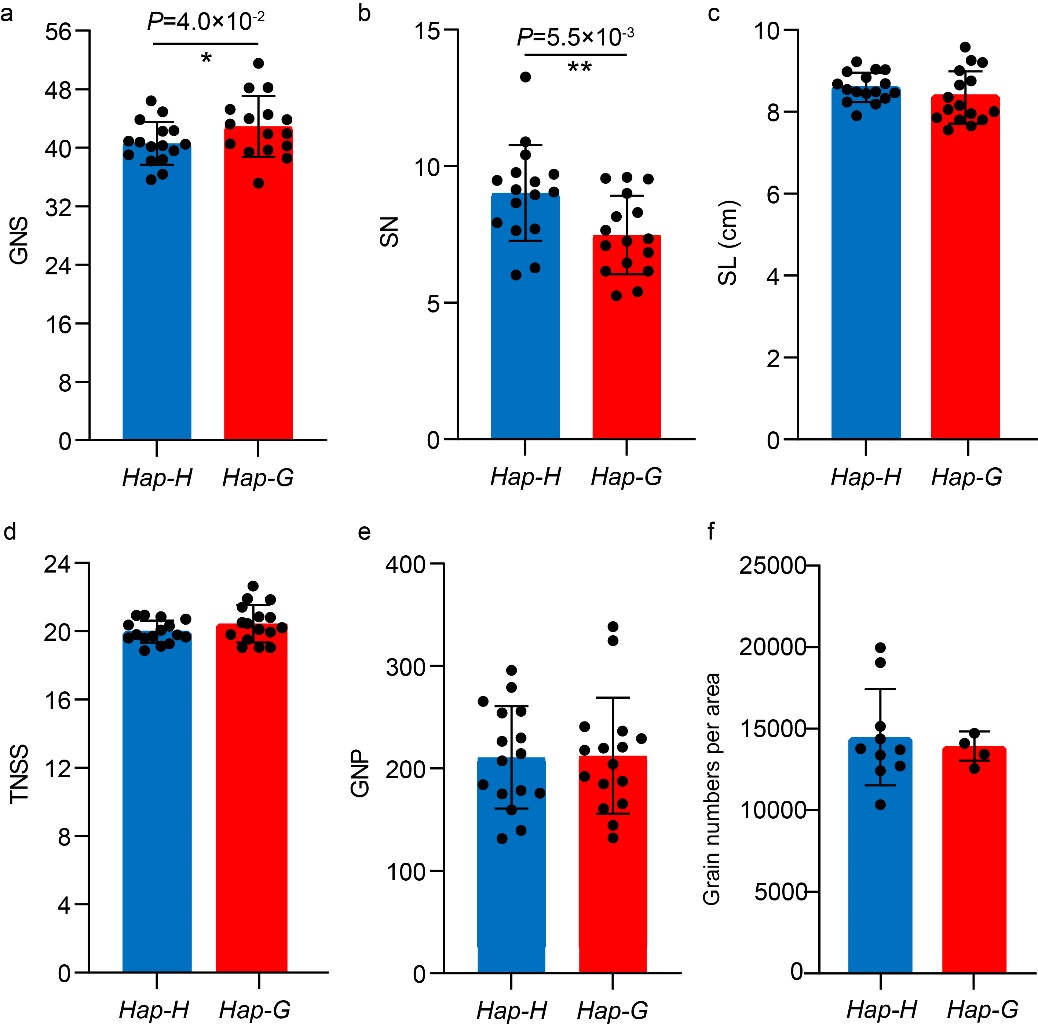


**Supplementary Figure 20. Aggregation of favorable haplotypes of *TaCYP78A3/5/16/17-Ap* affected yield-related traits.**

(a-f) Statistical analysis of GNS (a), SNPP (b), SL (c), TNSS (d), GNP (e), and grain numbers per area (f) of *Hap-H* and *Hap-G* accessions at 16 environmental sites, respectively (n=15). *Hap-H*: Genotypes with zero favorable haplotype of *TaCYP78A3/5/16/17-Ap* (*A3/5/16/17Ap-Hap II*); *Hap-G*: Genotypes with four *A3/5/16/17Ap-Hap II.* A point represents the average of phenotypic data from accessions with *Hap-H* and *Hap-G* at one environmental site. Values indicate means ± SE (n=20). **P* < 0.05, ***P* < 0.01 by Student’s *t*-test. GNS: Grain Number per Spike, SNPP: Spike Number per Plant; SL: Spike Length; TNSS: Total Number Spikelet per Spike; GNP: Grain Number per Plant.


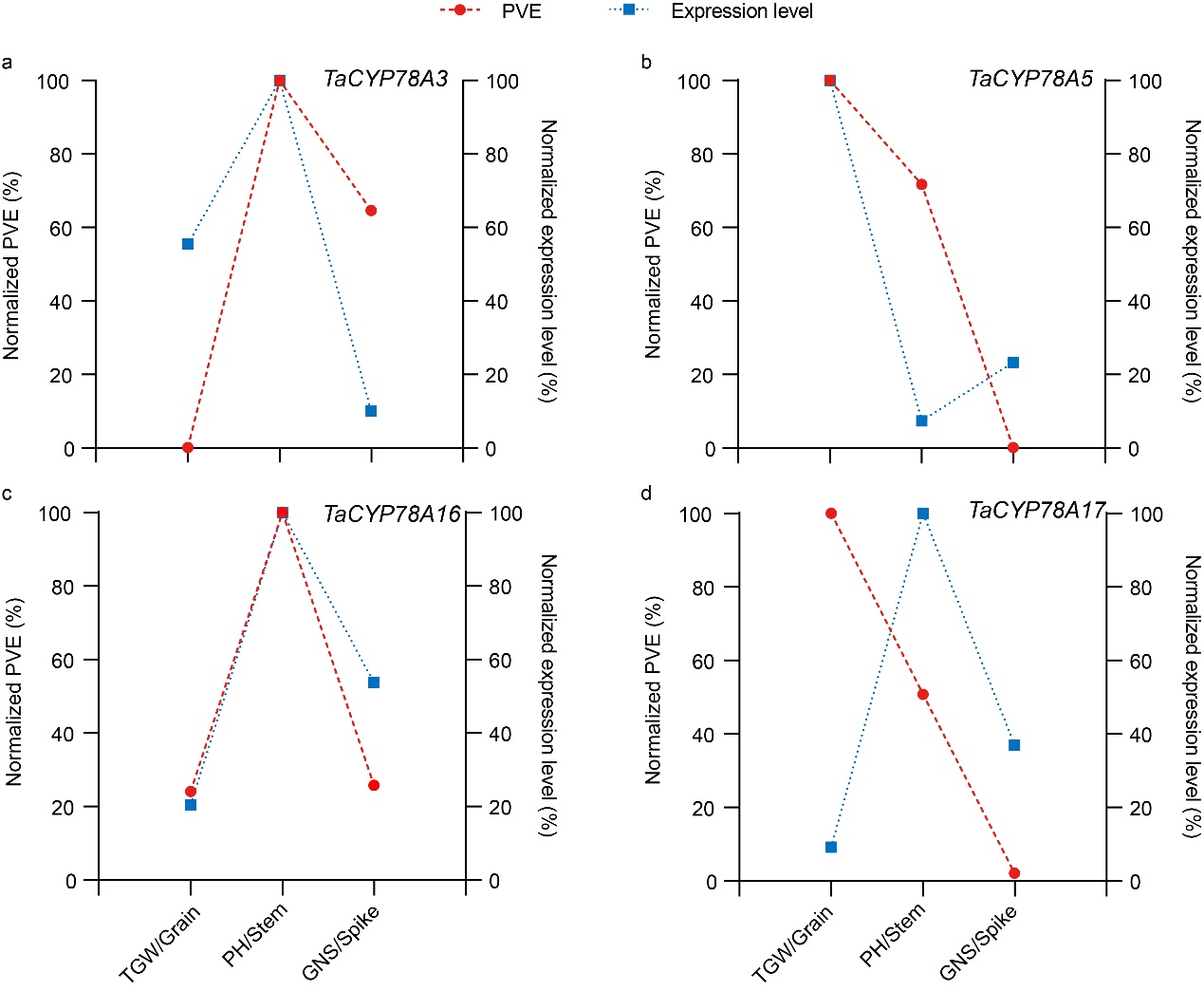


**Supplementary Figure 21 Compare the phenotypic variance explanation (PVE) and the expression levels of *TaCYP78A3/5/16/17-A* in different organs**

Phenotypic data were from 323 wheat accessions at 16 environmental sites. All data were normalized with the maximum value of expression levels or PVE.


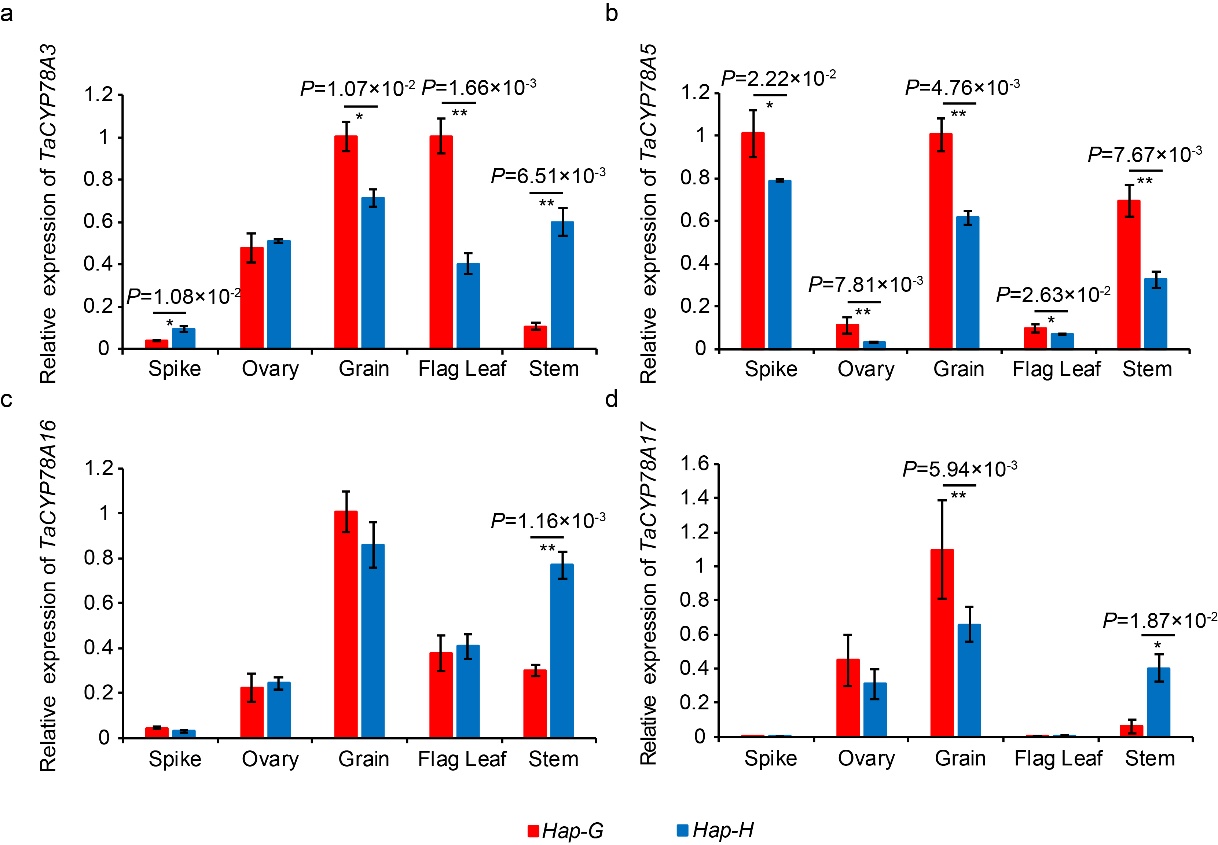


**Supplementary Figure 22. Comparison of the transcripts accumulation of *TaCYP78A3/5/16/17-A* in each organ from *Hap-H* and *Hap-G* accessions.**

(a-d) Quantitative real-time PCR (qRT-PCR) analysis of *TaCYP78A3-A* (a), *TaCYP78A5-A* (b) *TaCYP78A16-A* (c) and *TaCYP78A17-A* (d) expression level among organs from *Hap-H* and *Hap-G* accessions, respectively. *Hap-H*: Genotypes with zero favorable haplotype of *TaCYP78A3/5/16/17-Ap* (*A3/5/16/17Ap-Hap II*); *Hap-G*: Genotypes with four *A3/5/16/17Ap-Hap II.* *GADPH* (*TraesCS6B01G243700.1*) was used as a reference gene. Values indicate means ± SE (n = 3). **P* < 0.05, ***P* < 0.01 by Student’s *t*-test.


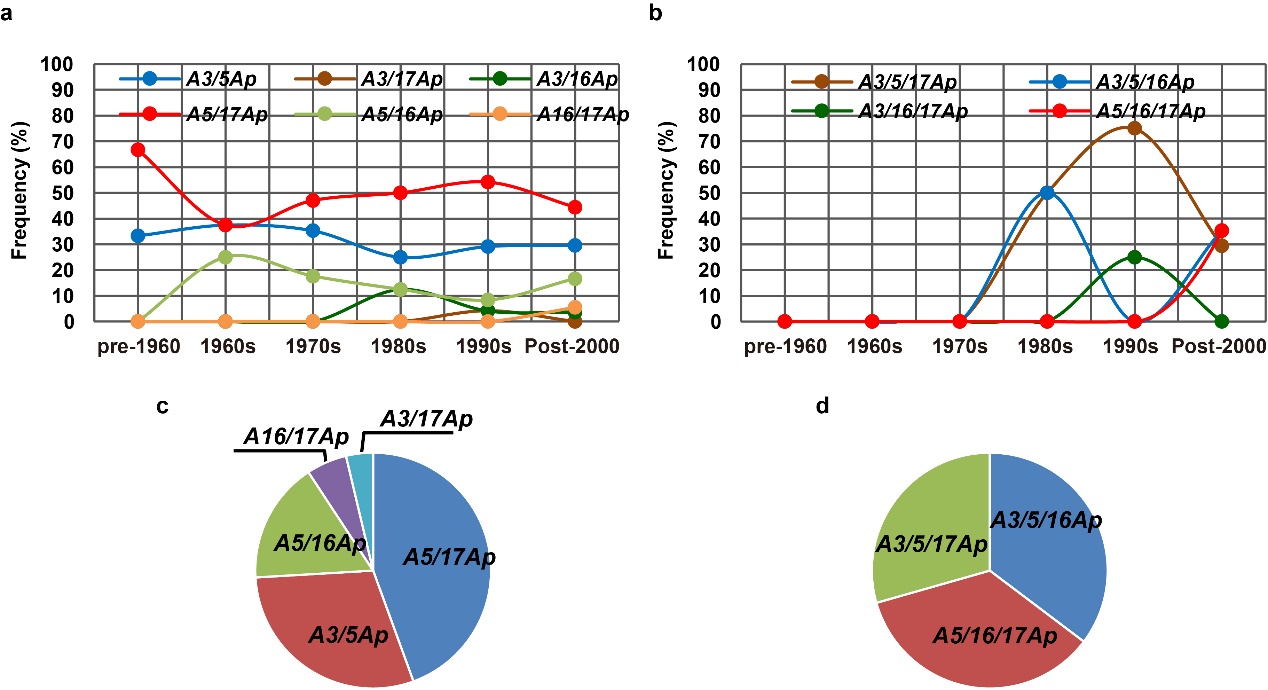


**Supplementary Figure 23. Frequency of favorable haplotype combinations of *TaCYP78A3/5/16/17-Ap* in the past 60 years of wheat breeding in China**

(a) The Frequency of different combinations containing two favorable haplotypes of *TaCYP78A3/5/16/17-Ap* in different years. (b) The Frequency of different combinations containing three favorable haplotypes of *TaCYP78A3/5/16/17-Ap* in different years. (c) The distribution frequencies of different combinations containing two favorable haplotypes of *TaCYP78A3/5/16/17-Ap* in Chinese Cultivars. (d) The distribution frequencies of combinations containing three favorable haplotypes of *TaCYP78A3/5/16/17-Ap* in Chinese Cultivars. Favorable haplotypes of *TaCYP78A3/5/16/17-Ap* named as *A3/5/16/17Ap* for simplicity, respectively.


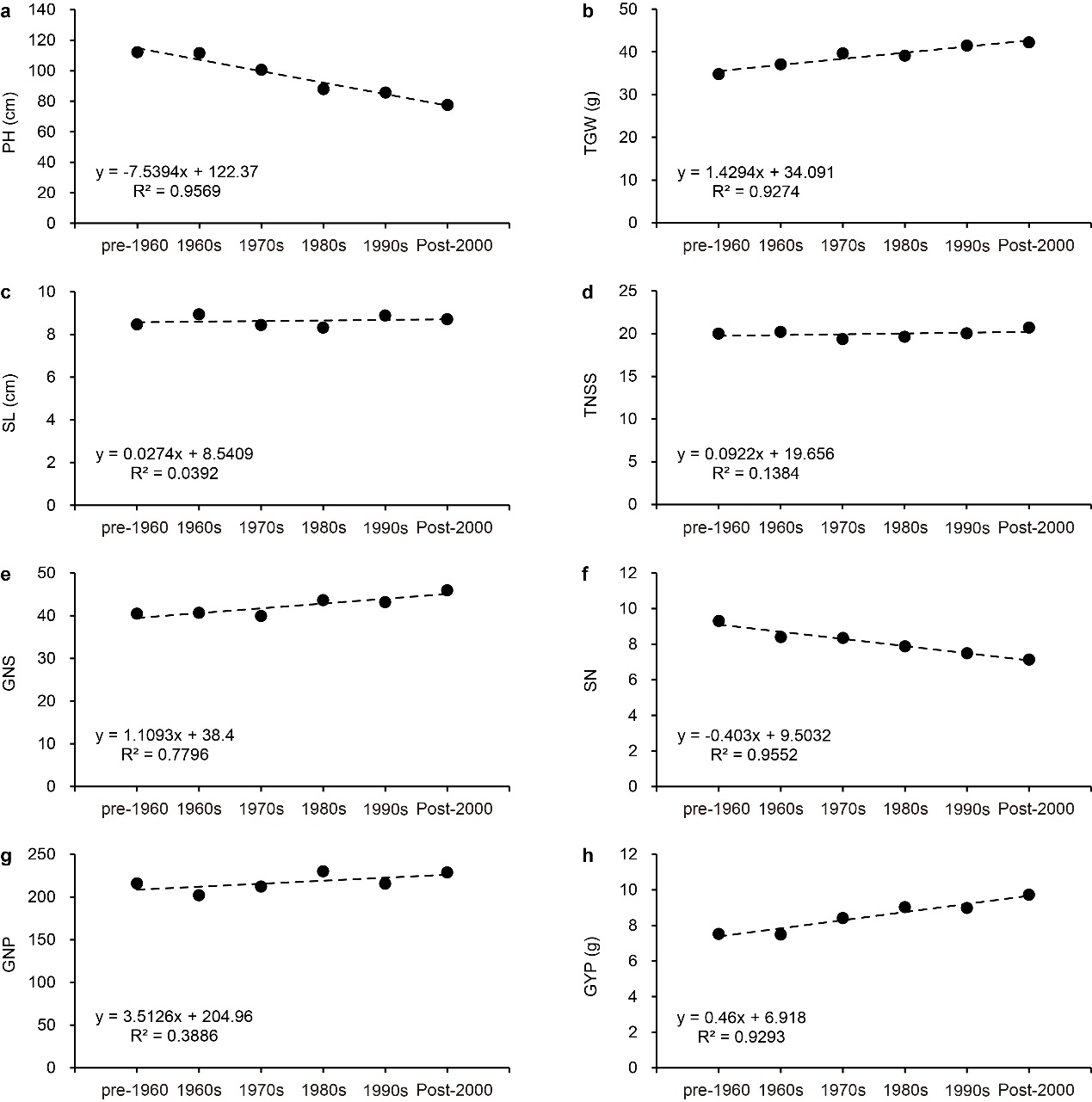


**Supplementary Figure 24. Variation trends of yield-related traits during wheat breeding in China.**

(a-h) Phenotypic data for yield-related traits was obtained from the average of 311 wheat accessions at 16 environmental sites. 14, 33, 47, 34, 60 and 123 accessions were released in pre-1960s, 1960s, 1970s, 1980s, 1990s and post-2000 respectively. PH: Plant Height; TGW: Thousand Grain Weight; SL: Spike length; TNSS: Total Number Spikelet per Spike; GNS: Grain Number per Spike; SNPP: Spikes Number per Plant; GNP: Grain Number per Plant; GYP: Grain Yield per Plant.

**References**

Chapple, C. (1998) Molecular-genetic analysis of plant cytochrome P450-dependent monooxygenases. *Annu Rev Plant Phys* **49**, 311-343.

Chi, Q., Guo, L.J., Ma, M., Zhang, L.J., Mao, H.D., Wu, B.W., Liu, X.L., Ramirez-Gonzalez, R.H., Uauy, C., Appels, R. and Zhao, H.X. (2019) Global transcriptome analysis uncovers the gene co-expression regulation network and key genes involved in grain development of wheat (Triticum aestivum L.). *Funct Integr Genomic* **19**, 853-866.

Shi, C.N., Zheng, Y.T., Geng, J.Y., Liu, C.Y., Pei, H., Ren, Y., Dong, Z.D., Zhao, L., Zhang, N. and Chen, F. (2020) Identification of herbicide resistance loci using a genome-wide association study and linkage mapping in Chinese common wheat. *Crop J* **8**, 666-675.
