# Supplementary material for: Pleiotropic phenotypic effects of the TaCYP78A family on multiple yield‐related traits in wheat
Source: Plant Biotechnol J. 2024 May 23;22(10):2694–708. doi: 10.1111/pbi.14385 (PMC11536447; doi:10.1111/pbi.14385)
Supplement: Supplementary file 3 — Appendix S1 Supplementary methods. [file PBI-22-2694-s001.docx]

**Supplementary methods**

**Construction of *TaCYP78As-A* overexpression vectors，mutation vectors and development of transgenic wheat lines**

The coding region of *TaCYP78A3/5/16-A* without TGA was inserted into the *pCAMBIA1304* vector via the restriction endonuclease *XbaI* and *BglII* to generated the overexpression vectors *pCAMBIA1304-UBI:TaCYP78A3/5/16-A*. The gdRNAs were designed on the CRISPRdirect website (http://crispr.dbcls.jp/) based on the *TaCYP78A3/5/17-A* coding region sequence, and inserted into the *pBUE411* vector via restriction endonuclease *BsaI* to generate the mutation vectors *TaU3p-TaCYP78A3/5/17-sgRNA*. The overexpression vectors *UBI::TaCYP78A3/5/16-A* and the mutation vectors *TaU3p-TaCYP78A3/5/17-sgRNA* were introduced into *Agrobacterium tumefaciens* strain *EHA105*, respecttively. All constructs were transformed into immature embryos 15 days after fertilization (DAF) of wheat (*T. aestivum* L.) cv. Fielder and its derivatives, using a modiﬁed *Agrobacterium*-mediated transformation method as previously described [1]. Positive transgenic plants were identified by leaf daubing with 0.2% glufosinate (BASTA), and transgenic lines were obtained by continuous self-crossing of single-locus transgenic plants and screening with Basta. Information on the constructs and primers used to generate the transgenic lines is shown in Supplementary Figure 13 and Supplementary Tables 7.

**RNA extraction and expression analysis**

Wheat tissue samples including the 1 mm size ovaries, 12 DAF grains, 3 cm long young spikes at booting stage and the top stem node, were kept at -80℃. Total RNA was extracted using the Steady Pure Plant RNA Extraction Kit (Accurate Biotechnology, Changsha, China), according to the manufacturer's instructions. The cDNA was synthesized using the Evo M-MLVRT Premix (Accurate Biotechnology, Changsha, China) according to the manufacturer’s protocol. Quantitative real-time PCR (qRT-PCR) was performed using the SYBR Green premix Pro Taq HS qPCR Kit (Accurate Biotechnology) on a CFX96 TM Real-time PCR Detection System (Bio-Rad, Hercules, USA). The wheat *β-actin* gene (TraesCS1A01G274400.1) was used as an endogenous control. The comparative threshold (Ct value) method was used to calculate the relative expression of each gene as previously described [2]. All data measurements were determined in at least three biological replicates for each sample.

**Statistical analyses**

Genotypic data based on genetic variations of *TaCYP78As-Ap* and phenotypic data of agronomic traits of the 323 wheat accessions at 16 environmental sites (E1–E16) over 3 years were analyzed using the TASSEL5.1 software (https://www.maizegenetics.net/tassel)[3]. PVE was performed as previously described [4]. Nucleotide diversities analysis (π) and Tajima’s D were performed using DnaSP5.10 (http://www.ub.edu/dnasp/) with a sliding window length of 100 and a step size of 10. Nucleotide diversity across species chromosomes was calculated using 33, 31, 13, 103, 43, 107 and 422 accessions each of Wild Emmer (WE), Domesticated Emmer (DE), *T. turgidum* L. var. durum, Chinese Landraces (CL), Chinese Cultivars (CC), Non-Chinese Landraces (NCL) and Non-Chinese Cultivars (NCC), respectively. Nucleotide diversity for each chromosome was derived from the average of nucleotide diversity in the promoter regions (2000 bp upstream of the ATG) of randomly selected 200 genes as previously described[5]. All data obtained were processed in Excel, and statistical analysis was performed using unpaired Student’s *t*-test at a signiﬁcance level of *P*-values <0.05 and <0.01.

**References**

1. Zhang S, Zhang R, Song G, Gao J, Li W, Han X, Chen M, Li Y, Li G. Targeted mutagenesis using the Agrobacterium tumefaciens-mediated CRISPR-Cas9 system in common wheat. BMC plant biology*.* 2018;18(1):302. <https://doi.org/10.1186/s12870-018-1496-x>.

2. Livak KJ, Schmittgen TD. Analysis of relative gene expression data using real-time quantitative PCR and the 2(T)(-Delta Delta C) method. Methods*.* 2001;25(4):402-8. <https://doi.org/10.1006/meth.2001.1262>.

3. Bradbury PJ, Zhang Z, Kroon DE, Casstevens TM, Ramdoss Y, Buckler ES. TASSEL: software for association mapping of complex traits in diverse samples. Bioinformatics*.* 2007;23(19):2633-5. <https://doi.org/10.1093/bioinformatics/btm308>.

4. Shi CN, Zheng YT, Geng JY, Liu CY, Pei H, Ren Y, Dong ZD, Zhao L, Zhang N, Chen F. Identification of herbicide resistance loci using a genome-wide association study and linkage mapping in Chinese common wheat. Crop J*.* 2020;8(4):666-75. <https://doi.org/10.1016/j.cj.2020.02.004>.

5. Caicedo AL, Williamson SH, Hernandez RD, Boyko A, Fledel-Alon A, York TL, Polato NR, Olsen KM, Nielsen R, McCouch SR, et al. Genome-wide patterns of nucleotide polymorphism in domesticated rice. Plos Genetics*.* 2007;3(9):1745-56. <https://doi.org/10.1371/journal.pgen.0030163>.
